# Structural analysis of the BisI family of modification dependent restriction endonucleases

**DOI:** 10.1093/nar/gkae634

**Published:** 2024-07-23

**Authors:** Katarzyna Szafran, Dominik Rafalski, Krzysztof Skowronek, Marek Wojciechowski, Asgar Abbas Kazrani, Mirosław Gilski, Shuang-yong Xu, Matthias Bochtler

**Affiliations:** International Institute of Molecular and Cell Biology, Warsaw, Poland; International Institute of Molecular and Cell Biology, Warsaw, Poland; Institute of Biochemistry and Biophysics, Polish Academy of Sciences, Warsaw, Poland; International Institute of Molecular and Cell Biology, Warsaw, Poland; International Institute of Molecular and Cell Biology, Warsaw, Poland; International Institute of Molecular and Cell Biology, Warsaw, Poland; Faculty of Chemistry, Adam Mickiewicz University, Poznan; Institute of Bioorganic Chemistry, Polish Academy of Sciences, Poznan, Poland; New England Biolabs, Ipswich, USA; International Institute of Molecular and Cell Biology, Warsaw, Poland; Institute of Biochemistry and Biophysics, Polish Academy of Sciences, Warsaw, Poland

## Abstract

The BisI family of restriction endonucleases is unique in requiring multiple methylated or hydroxymethylated cytosine residues within a short recognition sequence (GCNGC), and in cleaving directly within this sequence, rather than at a distance. Here, we report that the number of modified cytosines that are required for cleavage can be tuned by the salt concentration. We present crystal structures of two members of the BisI family, NhoI and Eco15I_Ntd (N-terminal domain of Eco15I), in the absence of DNA and in specific complexes with tetra-methylated GCNGC target DNA. The structures show that NhoI and Eco15I_Ntd sense modified cytosine bases in the context of double-stranded DNA (dsDNA) without base flipping. In the co-crystal structures of NhoI and Eco15I_Ntd with DNA, the internal methyl groups (G**5mC**NGC) interact with the side chains of an (H/R)(V/I/T/M) di-amino acid motif near the C-terminus of the distal enzyme subunit and arginine residue from the proximal subunit. The external methyl groups (GCNG**5mC**) interact with the proximal enzyme subunit, mostly through main chain contacts. Surface plasmon resonance analysis for Eco15I_Ntd shows that the internal and external methyl binding pockets contribute about equally to sensing of cytosine methyl groups.

## Graphical Abstract

**Graphical Abstract:**
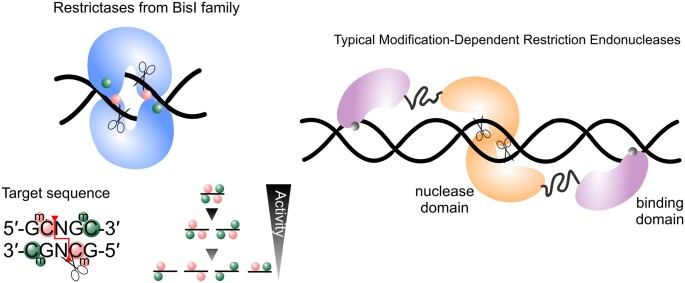

## Introduction

Canonical restriction-modification (R-M) systems target non-modified DNA, protecting host DNA by modifications, typically methylation (1). In response to these defense systems, some phages use methylated, hydroxymethylated, or otherwise modified DNA to escape restriction. It is thought that the emergence of phages with such DNA has driven the evolution of orphan modification dependent restriction endonucleases (MDREs) that are not accompanied by a methyltransferase (MTase) and target specifically modified DNA. Depending on whether the enzymes cleave within or outside a recognition sequence, they are classified as Type IIM or Type IV restriction endonucleases (2).

Most MDREs have separate modification sensing and catalytic subunits. The modification sensing domains typically belong to the PUA superfamily, named for the occurrence of the domain in PseudoUridine synthase and Archaeosine transglycosylase (3). The superfamily comprises the PUA domains in the strict sense (Interpro IPR002478) (4), the EVE domains (Interpro IPR002740) (5), the ASCH domains (Interpro IPR007374) (6), the SRA/YDG domains (Interpro IPR003105) (7), and according to some classifications, also the related YTH domains (IPR007275) (8). Members of PUA superfamily share a characteristic fold of five β-sheets forming a ‘pseudobarrel’ and have roles in nucleobase binding or modification. With the exception of the SRA/YDG domains, which were originally studied in the context of DNA methylation (9), the other PUA superfamily domains were first implicated in RNA nucleobase recognition or modification(4–6,10,11). Later, it became clear that EVE and YTH domains could also serve as modification sensors for DNA nucleobases (12,13). As DNA sensors, SRA and EVE domains sense modified cytosine bases (14,3), whereas YTH domains sense modified adenines (15). In all cases, the PUA superfamily domains extrude the DNA bases from double-stranded DNA, and scrutinize them in a dedicated pocket (9,14,16–18). Therefore, these domains sense methylation of a single modified DNA base. In addition to the PUA superfamily domains that flip the modified base, other modification sensing domains are the winged helix domains (Interpro IPR036390), found in DpnI (19) and many other MDREs (20), and the NEco domain, found at the N-terminus of EcoKMcrA (21). The non-PUA superfamily domains detect adenine or cytosine modifications in both DNA strands simultaneously, and they do so in the context of double-stranded DNA, without base flipping (19,22,23).

To form the MDRE, the modification sensing domains are combined with nuclease modules of the PD-(D/E)XK (24), HNH (His-Me, ββα-Me) (25,26) or GIY-YIG fold (27). The catalytic domains typically have no or very limited modification or sequence specificity by themselves. The enzymes cleave DNA at a distance of ∼10 base pairs (bps) from the modification, as observed for the endonucleases SRA-PD-(D/E)XK (MspJI) (28), PD-(D/E)XK-SRA (PvuRts1I) (29,30), SRA-HNH (TagI) (17,31), EVE-HNH (VcaM4I) (18) and NEco-HNH (EcoKMcrA) (22). Since the nuclease domains are dimers (or higher order assemblies of dimers), there are typically two modification sensing domains in the dimer. Cleavage is believed to be most efficient when two suitably spaced methyl/hydroxymethyl groups are present to direct cleavage (17,29). The DpnI family of modular MDREs is unusual, because the endonuclease domains in this family have independent sequence and modification specificity (19,23,27). In these cases, cleavage occurs within the recognition sequence of the catalytic domain (32). The modification sensor domain acts more like an effector domain in Type IIE restriction endonucleases (33), except that the sequence recognized by the modification sensor and catalytic domains is methylated.

The BisI and GlaI families of restriction endonucleases are specific for 5-methylcytosine (5mC) or 5-hydroxymethylcytosine (5hmC) containing DNA (34–36). They stand out from the other families of MDREs because they lack a separate methylation sensor domain, cleave in the immediate vicinity of the modified cytosine bases, and require 2–4 methylated or hydroxymethylated cytosines for DNA cleavage. Their (related) target sequences are either palindromic (GlaI, G5(h)mC↓G5(h)mC) (36), or nearly palindromic (BisI, G5(h)mC↓NG5(h)mC; Esp638I, R5mCN↓NGY, the cytosine opposite to G is also modified) (34,35). The cleavage patterns are consistent with the approximate symmetry of the targets, suggesting that the enzymes could be dimers. Bioinformatic analysis of the sequences shows that these enzymes belong to the PD-QXK subgroup (Mrr-like catalytic site) (37) of the PD-(D/E)XK MDREs with a αβββαβ fold core. However, sequence analysis alone was insufficient to elucidate the mechanistic basis of the preference for methylated or hydroxymethylated DNA in the family.

Here, we report a biochemical characterization of two members of the BisI family, NhoI from *Nitrolancea hollandica* and Eco15I from an *Escherichia coli* strain 541–15. We present the crystal structures of NhoI and Eco15I_Ntd (N-terminal catalytic domain of Eco15I) in the absence of DNA and in specific complexes with fully methylated GCNGC containing dsDNA. Both proteins are homodimeric, with each domain involved in target sequence recognition and catalysis. The crystal structures show that the modified cytosine bases are recognized in the context of double-stranded DNA, without base flipping. Protein-DNA binding and activity of NhoI and Eco15I_Ntd can be tuned by the salt concentration in the reaction buffer.

## Materials and methods

### Expression constructs

Amino acid sequences of wild-type proteins used in this study are shown in Supplementary Table S1. Full-length recombinant Eco15I was expressed from a pTYB1 based expression construct described previously (34). The boundaries of the catalytic domain were defined by limited proteolysis and mass spectrometry. The catalytic domain was then further truncated based on the secondary structure predictions using Yaspin (38) and JPRED (39) software (Supplementary Figure S1). The coding sequence for the truncated fragment was cloned with BamHI and XhoI restriction enzymes into a pET28a based expression vector so that the expressed protein acquired an N-terminal 6xHis-SUMO tag. The resulting construct is referred to as Eco15I_Ntd in further text. The NhoI gene was amplified by PCR from the previous construct (34) in pTYB1 vector and cloned between BamHI and XhoI restriction sites into pET28a plasmid containing N-terminal 6xHis-SUMO tag. Catalytic mutants of Eco15I_Ntd (Eco15I_Ntd_E49A, Eco15I_Ntd_D69A, Eco15I_Ntd_Q79A and Eco15I_Ntd_K81A) and NhoI (NhoI_H139A and V140_A) were generated by a Quick Change site-directed mutagenesis using primers stated in Supplementary Table S2. All inserts were sequenced together with tags to confirm that correct coding sequences were present in the plasmids.

### Protein expression and purification

Expression constructs of Eco15I, Eco15I_Ntd and NhoI (wild type and mutants) were transformed into *E. coli* ER2566 cells (NEB). All proteins were expressed in LB medium at 25°C upon induction with 0.5 mM isopropyl β-d-1-thiogalactopyranoside (IPTG). Protein production was induced at the OD_600_ of 0.7–0.8. Cells were harvested after 16–18 h by centrifugation and the pellets were stored at -20°C. Expression of the selenomethionine (SeMet) version of Eco15I_Ntd was performed in methionine auxotrophic *E. coli* B834(DE3) cells in a methionine-deprived medium supplemented with selenomethionine (Molecular Dimensions). Protein production was induced at the OD_600_ of 0.8.

Full-length Eco15I with C-terminal intein-CBD tag was selectively captured on a gravity flow chitin column (chitin resin, NEB S6651L). Upon intein cleavage induced with addition of 50 mM DTT the protein was eluted from chitin column and further purified with salt gradient on the HiTrap^®^ Heparin HP affinity column (GE Healthcare). Final purification step and buffer exchange were conducted on the Superdex^®^ S200 size exclusion chromatography column (GE Healthcare). NhoI and Eco15I_Ntd wild type and mutants were purified with affinity chromatography on the gravity flow Ni-NTA column (Qiagen). After elimination of the SUMO-tag upon Ulp1 cleavage proteins were subject to size exclusion chromatography on Superdex^®^S75 column (GE Healthcare), concentrated with the 3 kDa MWCO Amicon Ultra-15 centrifugal filter units (Merck) and stored in their respective buffers. Storage buffer for Eco15I and all Eco15I_Ntd variants consisted of 20 mM HEPES, pH 7.8, 50 mM NaCl, 2 mM DTT, 1 mM CaCl_2_; while the storage buffer for NhoI variants contained 20 mM HEPES, pH 8.0, 150 mM NaCl, 2 mM DTT, 1 mM CaCl_2_. Proteins were flash-frozen in their buffers supplemented with 15% (v/v) glycerol and kept at -80°C for long-term storage.

### Activity assay

Endonuclease activity of purified proteins was assayed with non-modified, fully methylated or fully hydroxymethylated DNA: either PCR products containing 550 bp (part of mBDNF gene; GenBank: AY057907.1, nt. 22904–23453) or dsDNA oligonucleotides listed in Supplementary Table S3. 30 ng (0.038 pmol) of PCR product or 10 pmol of oligonucleotide were incubated with different amounts of protein for 40 or 60 minutes at 37°C. Subsequently enzymes were inactivated for 5 minutes at 80°C or 20 min at -20°C. The reactions were performed in the buffers containing varying KCl concentrations (20–750 mM) containing 20 mM HEPES, pH 7.8, 1 mM DTT and 2 mM MgCl_2_. Results were visualized by 1 or 2% (w/v) agarose gel electrophoresis or native polyacrylamide gel electrophoresis (0.5 × TBE, 20% (v/v) acrylamide, 19:1 acrylamide:bisacrylamide) stained with GelRed (Biotium).

**Figure 1.**
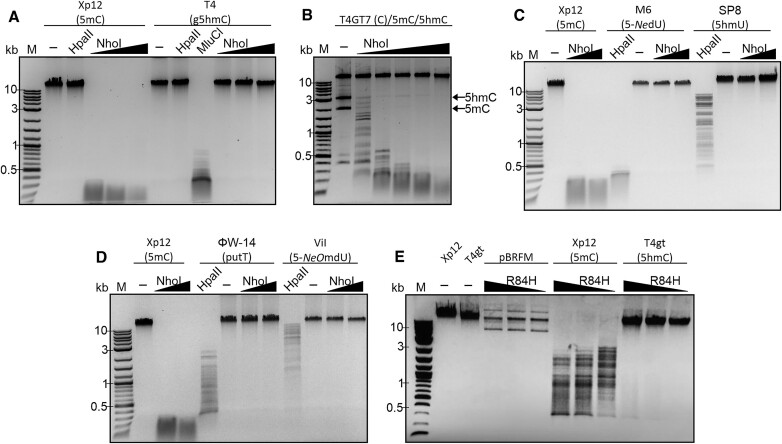
Digestion of DNA from various phages with substitution of canonical C (top) or T (bottom) bases. All digests were done with NhoI wild-type (**A–D**) or R84H mutant (**E**) in NEB buffer 2.1 at 37°C, for 1 h (T4GT7, Xp12, T4gt, T4 (WT)) or 1 h 20 min (SP8, ViI, M6, Phi-W14). The gels show representative data for at least three repeats. 350 ng of phage DNA were used except for the T4GT7 experiment, in which 0.2 μg of phage DNA was spiked with 5hmC (3 kb, 150 ng) and and 5mC-PCR product (2 kb, 150 ng) as a positive control. NhoI endonuclease (dimer) in panel A: 0.02–2 μg; in panel B: 0.2 ng–2 μg; in panels C and D: 0.2 μg and 2 μg. NhoI variant R84H in panel E: 0.02–2 μg protein. ‘—’ indicates the absence of enzyme. Note that pBRFM is a pBR322 derived plasmid that constitutively expresses the methyltransferase M.Fnu4HI, which methylates the inner C bases of the GCNGC recognition sequence of the BisI family of enzymes (34). Note also that 5hmU hypermodifications occur mostly at sites preceding G and are therefore not fully penetrant (59).

NhoI activity on phage DNA was tested in NEB buffer 2.1 (50 mM NaCl, 10 mM Tris–HCl pH 7.9, 10 mM MgCl_2_, 1 mM DTT, 100 μg/ml BSA) at 37°C, for 1 h (T4GT7-unmodified, Xp12, T4gt, T4 (WT)) or 1 h 20 min (SP8, ViI, M6, Phi-W14). 0.2 ng–2 μg of NhoI variants and 350 ng of phage DNA were used except for the T4GT7 experiment (Figure 1B), in which 0.2 μg of phage DNA were spiked with 5hmC- (3 kb, 150 ng) and 5mC-PCR product (2 kb, 150 ng) as the positive control for NhoI activity.

T4GT7 (40) phage was kindly provided by Geoff Wilson (NEB). Phage T4gt (shorthand for T4 αgt^−^ βgt^−^) (41) was kindly provided by Elisabeth Raleigh (NEB). Phages SP8, ViI, M6 and phi W-14 were kindly provided by Peter Weigele (NEB). Phage DNA was extracted by phenol-CHCl_3_ extraction (3×) and CHCl_3_ extraction (1×). DNA was precipitated by ethanol and salt.

### Electrophoretic mobility shift assay (EMSA)

The binding preferences of Eco15I_Ntd to various substrates were evaluated by EMSA. The forward strand of each oligonucleotide (listed in Supplementary Table S1) was labeled with radioactive [^33^P]-ATP using polynucleotide kinase (Thermo Scientific). Upon labelling and annealing with the reverse strand, DNA was purified with Mini Quick Spin Columns (Roche). 1 pmol of DNA was incubated for 30 min on ice with varying amount of Eco15I_Ntd (0.1–200 pmol) in a buffer containing 20 mM HEPES, pH 7.8, 50 mM KCl, 1 mM DTT and 1 mM CaCl_2_. Total sample volume was 10 μl. Either 10 pmol of unlabeled non-modified oligonucleotide of a cognate sequence or 10 ng of sheared salmon sperm DNA was added to each sample as a competitor DNA. 2 μl of 6x native loading solution (10 mM Tris–HCl, pH 7.5, 60% (v/v) glycerol, 0.03% (w/v) bromophenol blue) was added to each sample before loading on the native gel (0.5 × TB buffer, 10% (v/v) acrylamide, 19:1 acrylamide:bisacrylamide). Autoradiograms were scanned using a Typhoon scanner (GE Lifesciences).

### Crystallization

All proteins and protein-DNA complexes were crystallized at 18°C using the sitting drop vapor diffusion method. The reservoir buffer was mixed with an equal volume of protein or protein/DNA solution. NhoI was crystallized with 5′-CTG5mCAG5mCTC-3′/5′-GAG5mCTG5mCAG-3′ oligoduplex. Eco15I_Ntd was crystallized with 5′-AAG5mCAG5mCAA-3′/5′-TTG5mCTG5mCTT-3′ oligoduplex.

NhoI was mixed with methylated dsDNA in 1:1.1 molar ratio to a final concentration of 15 mg/ml in the storage buffer. The mixture was incubated on ice for 1 h and subsequently used to set up crystallization trials. Crystallization drops were set up using a Crystal Phoenix robot (Art Robbins Instruments). Drop sizes ranged from 0.4 to 1.2 μl. Crystals were obtained in the drops equilibrated against Morpheus D10 condition (0.1 M Tris–BICINE, pH 8.5, 20% (v/v) ethylene glycol, 10% (w/v) PEG 8000, 20 mM 1,6-hexanediol, 20 mM 1-butanol, 20 mM 1,2-propanediol, 20 mM 2-propanol, 20 mM 1,4-butanediol, 20 mM 1,3-propanediol). Crystals were harvested after 3 weeks from 1 μl drops and flash frozen in liquid nitrogen.

NhoI crystals were grown in crystallization drops with DNA containing 5hmC instead of 5mC, in the same conditions. Surprisingly, the diffraction analysis of these crystals showed that they did not contain DNA.

To obtain the crystals of Eco15I_Ntd in complex with dsDNA, the protein solution was mixed in 1:1.1 molar ratio with dsDNA to a final protein concentration of 23 mg/ml in the storage buffer. The mixture was incubated on ice for 1 h and subsequently used to set up crystallization trials. Crystals were harvested after 2 weeks from the 0.6 μl drops, equilibrated against 0.1 M HEPES, pH 6.5, 20 mM CaCl_2_, 35% (v/v) pentaerythritol ethoxylate and 10 mM NaSCN.

Eco15I_Ntd protein and its SeMet variant (25 mg/ml in storage buffers) were used for crystallization of the protein in the absence of DNA. Crystallization drops were set up using a Crystal Phoenix robot (Art Robbins Instruments). Drop sizes ranged from 0.4 to 1.2 μl. Crystals were harvested after 2 weeks from the drops, mixed with and equilibrated against 3 M NaCl, 0.1 M MES, pH 5.5 and 10 mM NaSCN. 5 M NaCl was used as a cryo-protectant to flash-cool the crystals.

### Structure determination

Diffraction data for crystals of Eco15I_Ntd alone, its specific complex with target DNA, and for the specific complex of NhoI with target DNA were collected at beamlines P11 and P13 of DESY, Hamburg. Diffraction data for the NhoI crystals that turned out not to contain DNA were collected at BESSY, Berlin. All diffraction data were processed using XDS (42) (Table 1 and Supplementary Table S4).

**Table 1.** Key parameters of the crystal structures solved in this work

	Eco15I_Ntd–DNA	Eco15I_Ntd	NhoI–DNA	NhoI
Space group	*P* 3_2_ 2 1	*P* 2_1_ 2_1_ 2_1_	*P* 2_1_ 2_1_ 2	*P* 1 2_1_ 1
Res. (Å)	2.33	2.60	1.81	2.65
*R*_pim_ (%)	2.59	1.45	2.09	4.71
*R*_work_ (%)	26.41	25.90	20.01	22.16
*R*_free_ (%)	27.16	28.43	24.24	27.05
PDB	8Q5O	8Q5M	8RPX	8Q5N

The complete crystallographic table is presented in the Supplement.

The diffraction data obtained for the NhoI–DNA complex could be indexed assuming an orthorhombic lattice. The extinctions on the reciprocal lattice axes and the translation function signals in the molecular replacement were consistent with the assignment of the crystals to the space group P2(1)2(1)2. The structure was solved by molecular replacement using PHASER (43) with a search model for the dimer generated by AlphaFold (44), which was subsequently rebuilt in ArpWarp (45,46) to improve the fit with the electron density. Inspection revealed that the asymmetric unit of the crystals contained two complete NhoI dimers with bound dsDNA, along with an additional monomer, and a single DNA strand. The protein monomer and the single DNA strand were completed to a protein dimer with bound dsDNA by crystallographic symmetry. In the crystal lattice, the NhoI–DNA dimers were stacked in spirals, with five dimers per turn. The contacts between adjacent dimers were very similar along the spiral. It was therefore very surprising that the quality of the electron density for the protomers varied greatly. The density was excellent for one complete dimer (chains A, B) and good for the dimer built by crystallographic symmetry (chain E). For the remaining dimer in the asymmetric unit, the density was acceptable for one of the protomers (chain D) but very poor for the other protomer (chain C). Initially, the poor density of one protein chain was attributed to broken rotational symmetry. Therefore, the structure was solved again, now in the lower symmetry space group *P*2(1) using molecular replacement. This choice of lower crystallographic symmetry seemed to improve the electron density for the (now two) problematic chains. However, as the refinement progressed, the density for the problematic dimers became similarly poor as the density for the single problematic protomer in the orthorhombic space group. Since broken rotational symmetry did not resolve the issue of poor density for one protomer, we searched for evidence of broken translational symmetry. However, we could not detect any systematic patterns of non-indexed reflections. Non-indexed reflections were concentrated in regions where ice rings would be expected, arguing against broken translational symmetry. Despite the low density, refinement was possible, resulting in *R*_free_ and *R*_cryst_ values typical for the resolution. The deposited pdb file has been annotated to draw attention to the unresolved crystallographic issues in the structure.

Crystals of NhoI alone belonged to the monoclinic space group *P*12(1)1 and contained one NhoI dimer in the asymmetric unit. The structure was solved by molecular replacement using PHASER (43), with the NhoI dimer from the protein–DNA complex structure used as the search model.

Crystals of the Eco15I_Ntd–DNA complex belonged to the trigonal space group *P*3(2)21. Although the space group of the crystals was *P*3(2)21, the Patterson symmetry was close to 6/*mmm*. The intensity statistics were consistent with almost perfect merohedral twinning. From the Patterson symmetry, it could be deduced that the twinning axis had to be a 2-fold axis of the alternative space group *P*3(2)12, corresponding to twinning operator -h, -k, l. To solve the structure, multiple search models of the Eco15I dimer were generated using AlphaFold (44). Among these, the most promising ones were selected based on MOLREP (47) scores, and then used for molecular replacement in PHASER (43). As the twinning fraction was close to 0.5, a model-independent de-twinning of the data was not possible, and model-dependent de-twinning, as implemented in PHENIX (48) had to be used.

Crystals of the Eco15I_Ntd without DNA belonged to the orthorhombic space group *P*2(1)2(1)2(1) for the protein with standard methionine residues, and to the monoclinic space group P12(1)1 for its selenomethionine variant. In retrospect, these structures could have been solved by molecular replacement using any of the other structures as a search model. However, as the diffraction data of the Eco15I_Ntd without DNA become available before the other datasets, these models were not yet available. Instead, we initially used a search model generated using the RAPTOR web server (49). With this search model, we initially solved the structure of the monoclinic crystal form. After some manual improvement of the model, the resulting structure could then be used to interpret the orthorhombic form. In the end, only the structure of Eco15I_Ntd with regular methionine residues was further refined, and the structure of the selenomethionine variant was used only for model validation.

Manual model building was done in COOT (50). For refinement, we used REFMAC (51) in CCP4 (52) or PHENIX (48). The parameters of the diffraction data and the final refinement statistics are listed in Table 1 and Supplementary Table S4. The data were deposited in the Protein Data Bank under the following accession codes: 8Q5M, 8Q5N, 8Q5O, 8RPX.

### Binding affinity measurements

Binding affinities of Eco15I_Ntd and Eco15I_Ctd to dsDNA were measured with surface plasmon resonance (Biacore S200). Analyzes were performed in the running buffers (20 mM HEPES, pH 7.8, 1 mM CaCl_2_, 1 mM DTT, 0.05% (v/v) Tween-20) containing 50 or 150 mM KCl at 25°C. Annealed double-stranded oligonucleotides (listed in Supplementary Table S3) with a single biotin on 5′ end at concentration of 2 nM were immobilized on SA sensor chips (coated with streptavidin, GE Healthcare) at 5 μl/minute flow rate for 1 min. It resulted in immobilization levels of 20–30 RU. The assays were performed in multicycle mode at 30 μl/min flow rate. Each analyte concentration was injected for 5 min followed by a 10 min dissociation phase, 1 minute regeneration with 1 M NaCl and 3 mM EDTA at 30 μl/min was performed between injections. Interaction affinities were determined by fitting of response versus concentration plot to basic steady state affinity model in the Biacore S200 Evaluation Software.

## Results

### Choice of BisI family enzymes for our studies

In this study, we focus on NhoI and Eco15I as representatives of the BisI family of restriction endonucleases. These two enzymes were chosen because they could be expressed and purified in better yields than BisI. NhoI is a typical BisI family member, Eco15I contains an additional domain at the C-terminus of the protein. We truncated the enzyme to the shortest active form of the catalytic domain (containing 173 amino acids) to increase the quality of crystals and refer to this truncated form as Eco15I_Ntd (Supplementary Figure S1). At the outset of the work, we expected that the C-terminal domain of Eco15I (called Eco15I_Ctd, containing 110 amino acids) would act as a modification sensitive effector domain, in analogy (but not homology) to the winged helix domain of DpnI. However, this hypothesis could not be corroborated as modification sensitivity is similar for full-length Eco15I and Eco15I_Ntd (Supplementary Figure S1C). Binding of the fully modified target sequence by Eco15I_Ctd could not be detected in an SPR experiment (Supplementary Figure S2). BLAST (53) searches with the C-terminal domain of Eco15I identified only Eco15I orthologues from other enterobacteria that were all annotated as hypothetical proteins. p*I* calculations indicate that the C-terminal domain is slightly acidic (calculated p*I* ∼5.6), which rather speaks against DNA binding. Although Eco15I_Ntd is similarly acidic, DNA binding is aided by the metal cations in the active site. For the C-terminal domain, there is no indication for metal binding sites that could promote DNA binding. Therefore, the function of Eco15I_Ctd remains unknown. Amino acid sequences of all used proteins are shown in Supplementary Table S1.

### Substrate specificity

Enzymes of the BisI family are known to cleave DNA with GCNGC target sequence, provided the sequence is multiply methylated or hydroxymethylated. Dense DNA methylation/hydroxymethylation in phages is relatively rare, except when the host or phage genomes encode frequent MTases (such as GpC, CpG and multi-specificity MTases). We therefore tested the activity of NhoI against various phages with high levels of DNA base modification. As reported earlier (34,35), NhoI did not cleave unmodified DNA from phage T4GT7 and digested methylated DNA faster than hydroxymethylated DNA (Figure 1B). DNA with glucosyl-5-hydroxymethylcytosine (g5hmC) from WT phage T4 (54) was not degraded by NhoI, indicating that a bulky glucosyl group could not be accommodated by NhoI (Figure 1A). We also did not observe digestion of DNA of phages that have non-canonical bases instead of T: the DNA of *Pseudomonas* phage SP8 containing 5-hydroxymethyluracil (5hmU) (55), of *Salmonella* phage ViI (Vi1) containing 5-(2-aminoethoxy) methyldeoxyuridine (5-*N*e*O*mdU) (56), of *Pseudomonas* phage M6 containing 5-(2-aminoethyl) deoxyuridine (5-*N*edU) (56), and of *Delftia* phage phi W-14 (ΦW-14), containing α-putrescinylthymine (putT) (57,58) (Figure 1C, D). We therefore concluded that none of the bulky thymine analogues could substitute for 5mC or 5hmC in the recognition sequence and that g5hmC-modified DNA was completely immune to cleavage (Figure 1).

While the bulky thymine analogues could not substitute for 5mC, thymine itself could, at least *in vitro*. Eco15I_Ntd digested DNA with up to two T bases replacing 5mC bases in the recognition sequence. DNA cleavage occurred regardless of the position (external or internal) of the substituted 5mC bases. It was also irrelevant whether the 5mC-substituted T bases were mis-paired with G bases or paired with T bases (Supplementary Figure S3A and Supplementary Table S3). Since the DNA cleavage reaction produced fragments of the expected size, and since cleavage was abolished for enzyme variants with active site modifications (Supplementary Figure S3C), the DNA cleavage had to be due to the activity of Eco15I_Ntd and could not be attributed to an unidentified contaminating endonuclease. However, EMSA assays performed in the presence of Ca^2+^ ions instead of Mg^2+^ ions (to promote DNA binding but not DNA cleavage) showed that replacement of 5mC by T drastically reduced the affinity of DNA for Eco15I_Ntd (Supplementary Figure S3D). We therefore concluded that the cleavage of DNA substrates with T bases instead of the 5mC bases by Eco15I_Ntd was likely an *in vitro* artifact caused by the high enzyme concentration and high enzyme/DNA ratio, and was unlikely to be relevant in cells.

### Tunability of the requirement for methyl groups by the salt concentration

**Figure 2.**
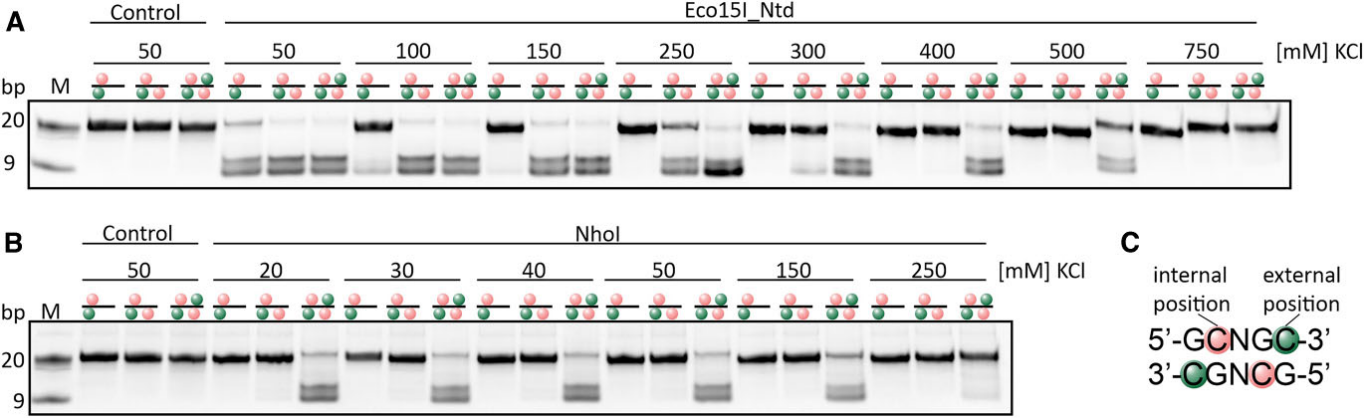
Salt dependence of DNA cleavage by Eco15I_Ntd (**A**) and NhoI (**B**). Oligonucleotides with 2–4 methyl groups within the enzyme recognition sequence were digested with Eco15I_Ntd or NhoI. Reaction products were analyzed on native PAA gels. The orientation of methyl groups is depicted schematically. Internal 5mC bases are marked in pink, and external 5mC bases are marked in green. The lack of a symbol indicates a non-methylated C. 10 pmol of DNA were incubated with 5 pmol of protein for 1 h at 37°C. Reaction conditions varied with salt concentration in the range of 20–750 mM KCl. Representative data for three repeats.

So far, the minimum number of methyl groups in the target sequence (typically 2 or 3, in some cases 4) was considered to be an intrinsic property of the enzymes from the BisI and GlaI families. We noted that the number of methyl groups that is required for DNA cleavage could be tuned by the salt concentration. In low salt conditions, Eco15_Ntd cleaved substrates with two or more methyl groups in the recognition sequence. Even in the case of the substrate with 2 methyl groups, their orientation did not affect digestion outcomes (Supplementary Figure S3B). As the salt concentration was increased, the ability to cleave the substrate with just two methyl groups was lost first. A further increase in salt concentration then also abolished the cleavage of the substrate featuring three methyl groups. At a very high salt concentration, even the duplex with fully methylated recognition sequence was no longer cleaved (Figure 2A). NhoI cleaved only the substrate with four methyl groups, for almost the entire range of tested salt concentrations (20–150 mM KCl). A slight cleavage of the substrates with less than four methyl groups was observed at high enzyme concentrations, well above physiological conditions (Supplementary Figure S4). At the highest salt concentration tested, even the cleavage of the fully methylated substrate was lost (Figure 2). These observations are biophysically reasonable, as the ionic interactions between the phosphodiester backbone and the endonuclease are attenuated at a higher salt concentration. Presumably, this loss of protein–DNA backbone interactions is not fully compensated by the increased strength of hydrophobic interactions of the substrate methyl groups with their binding pockets in the enzyme. Hence, more methyl groups are required in high salt than in low salt to achieve sufficient affinity between protein and DNA for productive DNA cleavage.

### Properties of the methyl binding pockets

**Figure 3.**
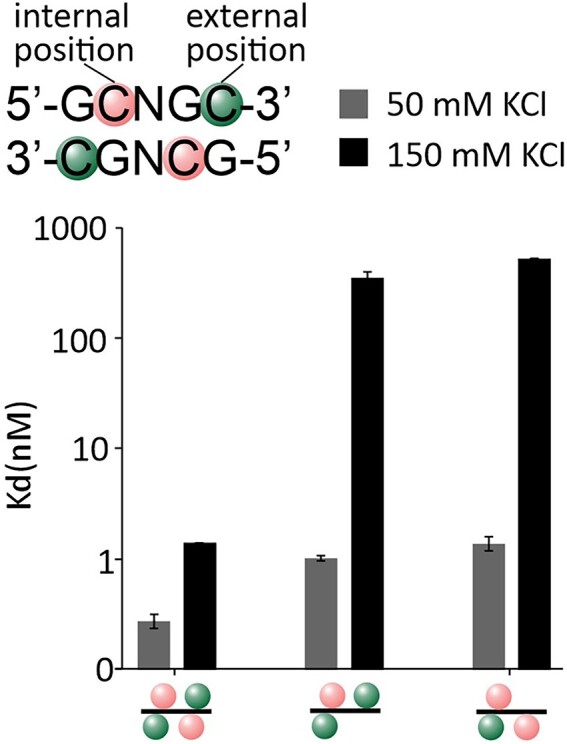
Salt dependence of DNA binding by Eco15I_Ntd. Binding affinity for substrates with three or four methyl groups was measured by surface plasmon resonance in the presence of Ca^2+^ ions to prevent the DNA cleavage The orientation of the methyl groups is depicted schematically. Internal 5mC is marked in pink, and external 5mC is marked in green. The fitting curves from the experiment are available in the Supplementary Figure S5. Error bars represent the standard error of the fit.

The nearly palindromic (pseudopalindromic) nature of the target sequence of the BisI family, and dimeric nature of most PD-(D/E)XK restriction endonucleases suggested that the enzymes have one type of methyl binding pocket for the 5′ (or internal, G**5mC**NGC) methyl groups of the target sequence, and another type of pocket for the 3′ (or external, GCNG**5mC**) methyl groups of the target sequence, as later confirmed by X-ray crystallography. We used surface plasmon resonance (60) to measure the binding of Eco15I_Ntd to dsDNA targets differing only in the number and/or position of methyl groups (Figure 3). To avoid the confounding influence of DNA cleavage on the kinetics, experiments were carried out in the presence of Ca^2+^ which prevents catalysis. Such conditions are expected to result in slightly tighter binding than would occur in the presence of catalytic Mg^2+^ (61). Reliable fitting of DNA affinity was only possible for recognition sequences with four or three methylated cytosines. For the substrate with two methylated cytosines, the apparent affinity was above 10 μM. However, quality problems with the sensograms prevented us from performing experiments at concentrations of Eco15I_Ntd above 20 μM, which would be required to properly estimate *K*_D_ for such weak interactions (data not shown).

Affinity between Eco15I_Ntd and its target was highest when the target was fully methylated with four methyl groups and decreased with decreasing number of methyl groups. As the targets with three and four methyl groups are sufficient to characterize the properties of both binding pockets, we focused on these substrates. In low salt, the omission of one methyl group reduced the affinity by less than an order of magnitude. In higher salt, binding constants were lower overall, and the omission of a methyl group had a more drastic effect, reducing the affinity by two to three orders of magnitude. Overall, the data indicate that external and internal methyl binding pockets contribute similarly to binding of DNA (Figure 3, Supplementary Figure S5).

### Crystals and structure determination

Crystals of NhoI and Eco15I_Ntd could be grown in the absence of DNA, and as specific complexes with tetra-methylated G5mCNG5mC DNA with Ca^2+^ cations replacing the Mg^2+^ cofactor. Crystals grown in the absence of DNA contained either an NhoI dimer, or two Eco15I_Ntd dimers in the asymmetric unit. Crystals that were grown in the presence of DNA contained 2$\frac{1}{2}$ NhoI dsDNA complexes, or one Eco15I_Ntd dsDNA complex in the asymmetric unit. All crystal structures were solved by molecular replacement and refined using REFMAC (51). Data collection and refinement statistics are summarized in Table 1 and Supplementary Table S4. As the crystals of the specific NhoI DNA complex diffracted far better than other crystals, the following structural description focuses largely on this complex. The other structures are discussed in comparison to this reference structure.

### Overall structure of NhoI and Eco15I_Ntd

**Figure 4.**
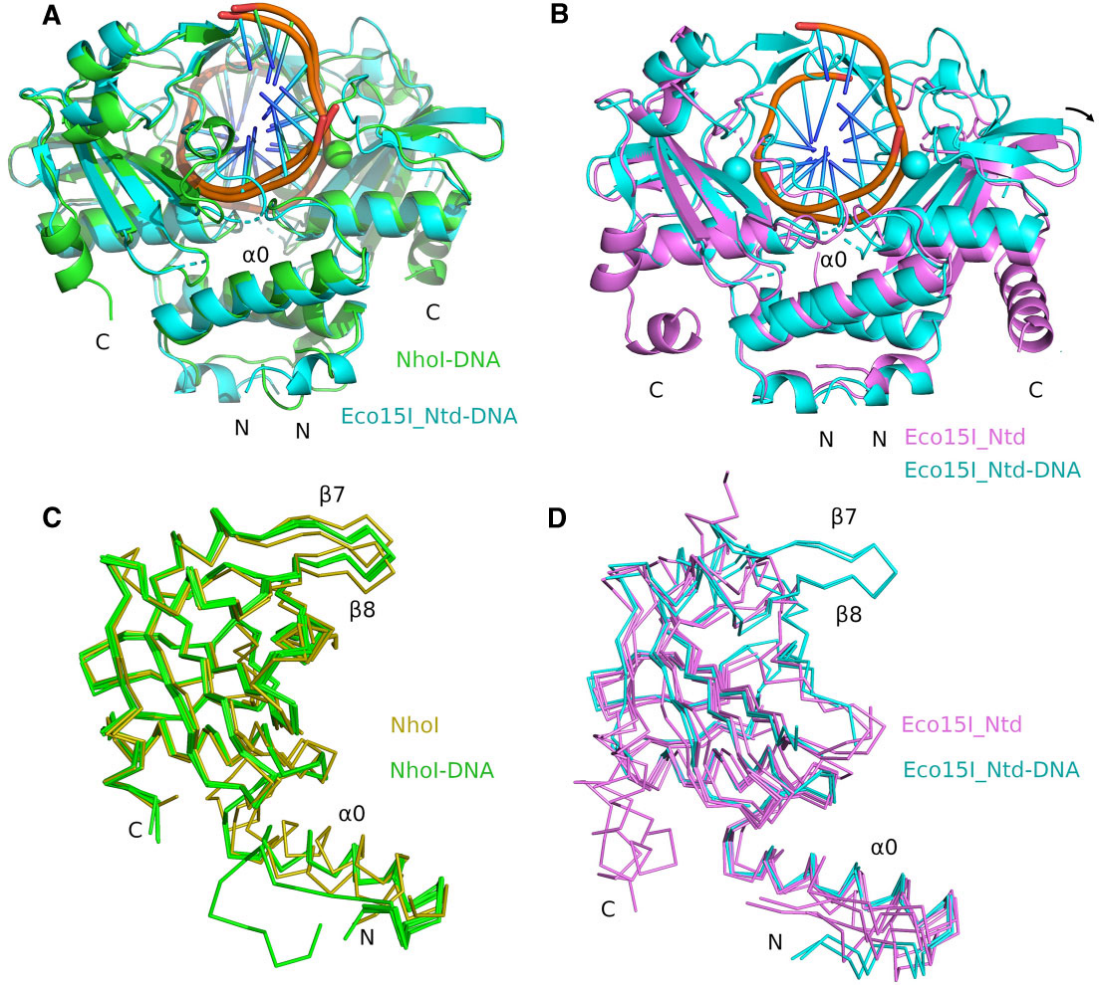
Comparison of BisI family structures: (**A**) Overlay of the specific DNA complexes of NhoI (green) and Eco15I_Ntd (cyan). (**B**) Overlay of the structures of Eco15I_Ntd with (cyan) and without (purple) target DNA. For panels A and B, the superposition operator was calculated based on the protomers on the left and structures are shown in ribbon representation. (**C**) Overlay of all subunits of NhoI, colored without (yellow) or with (green) DNA. (**D**) Overlay of structures of Eco15I_Ntd without (purple) and with (cyan) DNA. Structures in panels C and D are shown as Cα-traces.

In all crystals, Eco15I_Ntd and NhoI formed dimers. In the crystal structures of NhoI and Eco15I_Ntd with DNA, the dimerization and DNA binding modes are very similar (Figure 4). In the ‘standard’ orientation of Figure 4A when the dimer is oriented so that the two-fold symmetry axis runs vertically and the contact α0-helices are placed at the bottom, there is a large channel for DNA. This channel is delimited at the bottom by the linker region between α0 and α1. In the co-crystal structures with bound DNA, the channel is closed on top by the hairpins of β-strands β7 and β8. Interactions with DNA occur primarily in two regions. The linker region between α0 and α1 contacts the DNA from the minor groove side. Strand β3 of the core motif, which carries the catalytic QXK motif, comes closest to the phosphodiester backbone of the DNA and strand β4 approaches the DNA from the major groove side (Figure 4A and B).

In the Eco15I_Ntd crystal structure without DNA, the relative orientation of the protomers is very similar to the orientation seen in the DNA complex. The region of β-stands β7 and β8 is disordered, so that DNA binding channel is open from the ‘top’, presumably to allow the enzyme to load onto DNA (Figure 4D). In the NhoI crystal structure without DNA, the dimer adopts a more open conformation overall, primarily due to a different main chain conformation immediately downstream of the dimerization helix α0. However, the hairpin of β-stands β7 and β8 is much better ordered, likely because of crystal contacts to adjacent molecules (Figure 4C). Together, the data suggest that NhoI and Eco15I_Ntd can latch onto DNA thanks to an opening of the DNA binding cleft, and due to flexibility of the β-hairpin that closes the DNA binding cleft at the top.

### Detailed protomer fold

**Figure 5.**
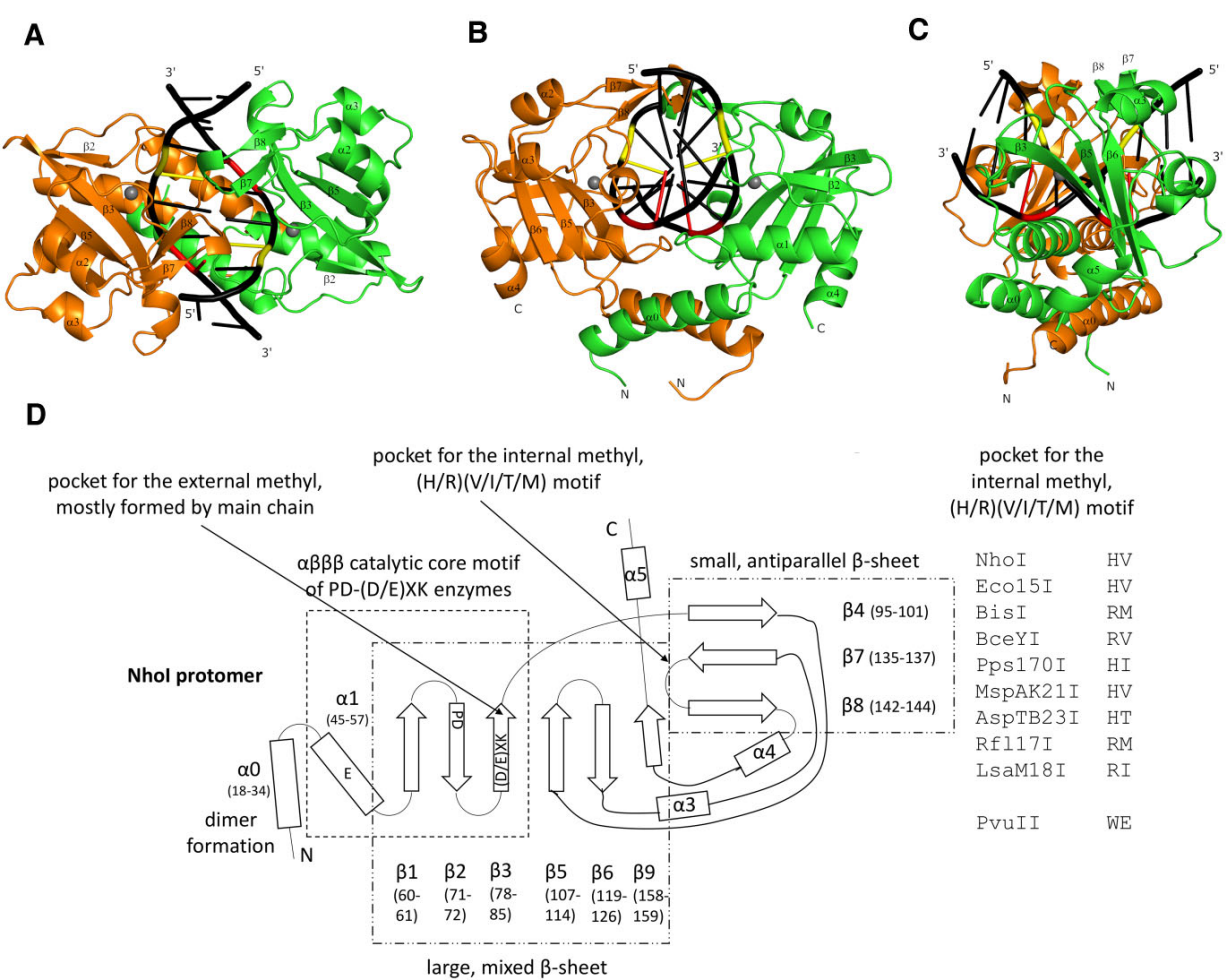
Overall NhoI fold. (**A–C**) Ribbon representation of the NhoI–DNA complex in different orientations. Subunits are colored in green and orange, respectively. Grey balls represent the Ca^2+^ ions in the complex that substitute for the Mg^2+^ ions in the active complex. (**D**) Schematic representation of the NhoI protomer fold. The diagram is based on manual inspection. Residue ranges for secondary structures are based on the very restrictive dssp criteria for secondary structure. The active site motifs are given in the canonical form as ‘PD’ and ‘(D/E)XK’. The ‘PD’ motif is ‘WD’ in NhoI and ‘AD’ in Eco15I. The ‘(D/E)XK motif is ‘QVK’ in NhoI and ‘QIK’ in Eco15I. The inset shows two columns of the multiple sequence alignment for the BisI family representing the region of the binding motif for an internal methyl group. The fold diagram applies equally to Eco15I_Ntd, albeit with offsets in the residue numbering.

Canonical PD-(D/E)XK restriction endonucleases have a core motif of an α-helix (termed here α1), followed by three consecutive antiparallel β-strands (β1, β2 and β3) (62). As expected, this core motif is present in NhoI and Eco15I_Ntd. In both enzymes, there is another α-helix (α0) present upstream, which contributes most to the dimer contacts. As in the majority of PD-(D/E)XK restriction endonucleases, there is also a considerable bulk downstream of the core motif. Altogether, the structure can therefore also be described as formed by two N-terminal α-helices (α0 and α1), a large mixed β-sheet (strands β1, β2, β3, β5, β6, β9), another antiparallel β-sheet formed from insertions in the large mixed β-sheet (β4, β7, β8), and two helices close to the C-terminus (α3 and α4) (Figure 5).

Both BLAST (63) and DALI (64) structural comparisons show that the closest relative of Eco15I and NhoI among the canonical Type II restriction endonucleases is PvuII, which shares most of the above-described features. PvuII is a blunt-end cutter, while the BisI family cuts DNA producing single base 5′-overhangs. As PvuII and the BisI family enzymes cleave with similar overhangs (0 and 1 bases), it is therefore not surprising that their dimerization modes are also similar.

### Active site

**Figure 6.**
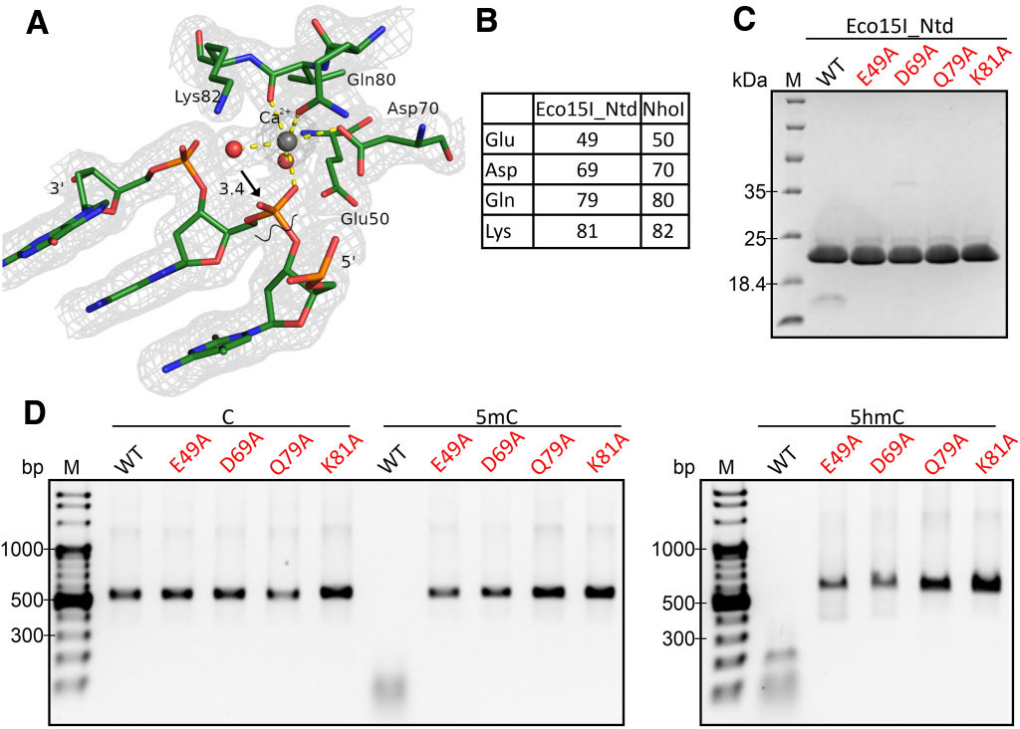
Active site and confirmation of the role of active sites residues. (**A**) Structure of the active site of NhoI. As crystallization was done in the presence of Ca^2+^, which does not support catalysis, the DNA is non-cleaved, and the density for it is continuous. The composite omit map is contoured at 1 σ, except for the metal ion which is contoured at 3 σ. Coordination is octahedral (yellow dotted lines). As in other cases with Ca^2+^ in the active site,a solvent molecule is almost poised for nucleophilic inline attack on the scissile phosphate (black arrow), except that the distance is slightly too large for the reaction to proceed. The bond that would be cleaved by the nucleophilic attack is marked by the wobbly line. A very similar active site region, albeit at lower resolution, is also seen in the crystal structure of Eco15I_Ntd with DNA. (**B**) Correspondence of active site residues in NhoI and Eco15I_Ntd (**C**) Coomassie stained SDS-PAGE gels of the enzymes used for activity assays. (**D**) Activity assays of Eco15I_Ntd wild type and mutants on DNA substrate containing four C, 5mC or 5hmC bases in the recognition sequence. 60 ng DNA was incubated for 1 h at 37°C with 10 pmol of enzyme. Representative data for three repeats.

Canonically, one expects the active site of PD-(D/E)XK endonucleases to be composed of an acidic residue (typically glutamate) in the α-helix of the catalytic core motif, of an aspartate at the N-terminal end of the second β-strand that is part of the PD-motif, and of an aspartate or glutamate and lysine that together from the (D/E)XK motif of the moniker PD-(D/E)XK designation. As in many other restriction endonucleases, the PD-motif can be varied. In NhoI and Eco15I, the motif is WD and AD, respectively. More variation in the amino acid upstream of the D is observed in the entire BisI-family, but the catalytic aspartate is conserved, as expected (Supplementary Figure S6, marked in blue). Like the PD motif, the original (D/E)XK motif is also known to vary. Often, the acidic aspartate or glutamate residue can be replaced by asparagine or glutamine, which are equally suited as metal chelating residues. The catalytic motifs in NhoI and Eco15I are QVK and QIK, respectively. A single Ca^2+^ ion from the buffer is present in the active sites of NhoI and Eco15I_Ntd in complex with DNA (Figure 6A and B). As expected, this Ca^2+^ ion is coordinated by the aspartate of PD and the glutamine of QVK. In some asymmetric unit protomers, a very clear water molecule density, geometrically poised for an inline attack on the scissile phosphate is also present, except that the distance is too large for catalysis to proceed (Figure 6A). As expected, the DNA electron density is continuous, indicating that the DNA is non-cleaved, as in other DNA-PD-(D/E)XK restrictase co-crystal structures obtained in the presence of Ca^2+^. However, the metal coordination is not fully canonical. Usually, a glutamate from the core motif α-helix (α1) would be expected to participate in metal coordination. In the NhoI crystal structure, this is not always the case. The glutamate (Glu50 in NhoI) comes close to the metal, but participates in the active site metal ion coordination only in some protomers in the asymmetric unit. As the NhoI–DNA crystals were obtained relatively late in the project, the consequence of the loss of active site residues was tested for Eco15I_Ntd. These results should be directly transferrable to NhoI and other BisI family members due to their active site residue conservation (Figure 6B). Like the wild type, all variant proteins could be purified to apparent homogeneity (Figure 6C). Activity analysis showed that all residue alterations, including the Eco15I_Ntd equivalent of Glu50, abolished the enzyme activity (Figure 6D).

### Sequence recognition of the modified DNA

The DNA in the NhoI–DNA complex is bound in both orientations that are compatible with the 2-fold symmetry of the enzyme. This does not compromise the electron density for the base pairs that follow the two-fold symmetry, i.e. of the external and internal G:5mC pairs. However, it somewhat blurs the density for the central symmetry breaking base pair, and for the flanking base pairs that do not follow the 2-fold symmetry. Due to the symmetry, it suffices to characterize one half-site of the recognition sequence and the central base pair. In the following, we describe the relation between a methyl group (or 5mC base, or the DNA strand containing the 5mC base) and a protein subunit as proximal, if the subunit would cleave the DNA strand of this methyl group, and as distal otherwise.

**Figure 7.**
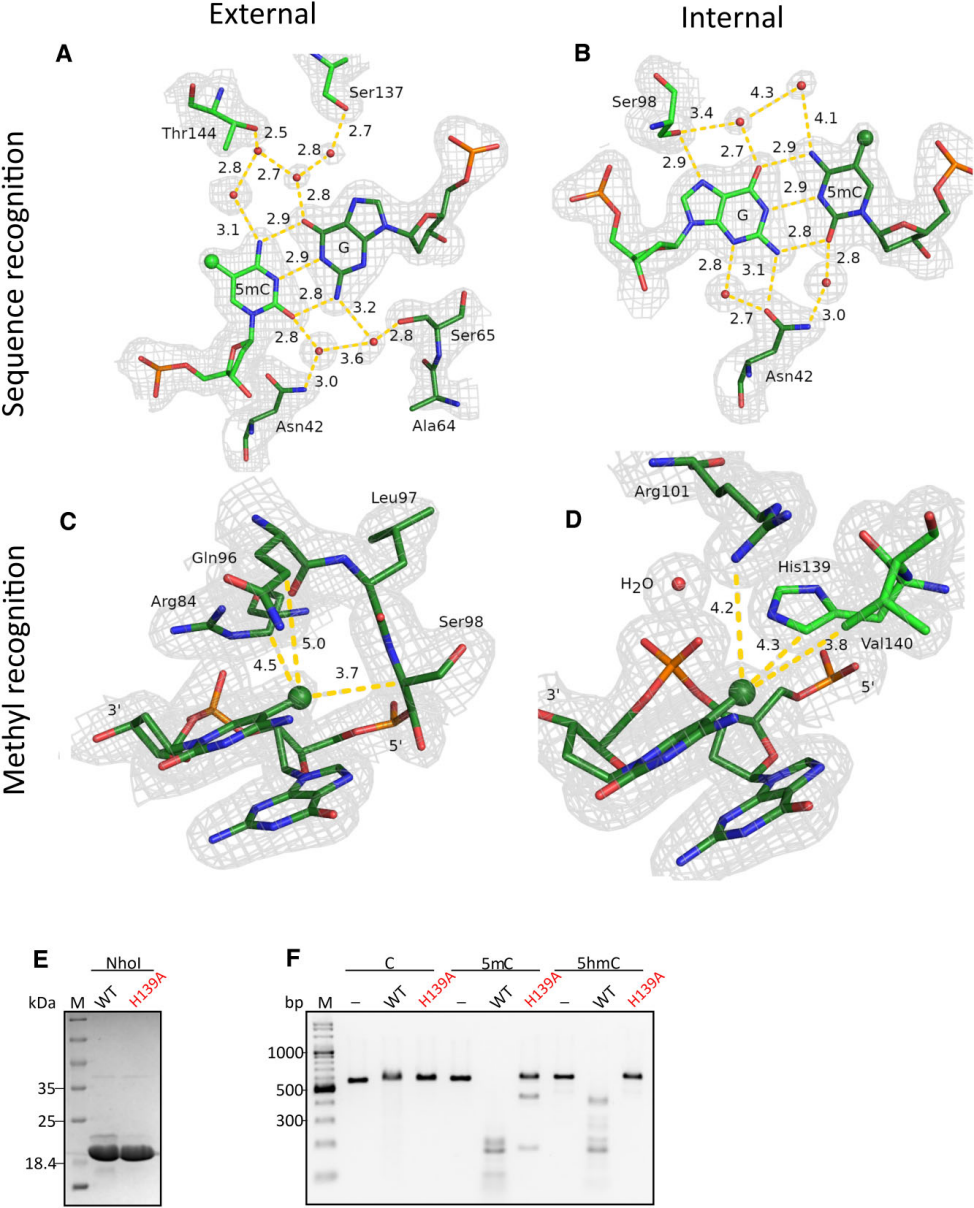
Sequence and 5-methylcytosine (5mC) recognition. Sequence recognition of the (**A**) external and (**B**) internal base pairs, and 5mC recognition of the (**C**) external and (**D**) internal methyl groups by NhoI. Methyl groups are emphasized by their representation as a ball. Residues from one subunit and the proximal DNA strand are in dark green, residues from the other subunit and its proximal DNA strand are in light green. The composite omit density has been contoured at 1 σ. (**E**) Coomassie stained SDS-PAGE gels with WT NhoI and NhoI variant H139A used for activity assays. (**F**) activity assays on DNA substrate containing C, 5mC or 5hmC within target sequence. 60 ng DNA was incubated for 1 h at 37°C with 10 pmol of enzyme. Representative data for three repeats.

Overall, both the external and internal 5mC bases of the recognition sequence are recognized surprisingly indirectly (Figure 7A and B, respectively). The external 5mC, and the paired guanine, do not form a single direct hydrogen bond with the protein. Instead, there are only water mediated hydrogen bonds from the proximal subunit on the major groove side (Thr144, Ser137), and from the distal subunit to the minor groove side (Asn42, Ser65). Recognition of the internal 5mC is more stringent, with a purine selecting hydrogen bond from the Ser98 (partially conserved, Supplementary Figure S6) to the N7 of the paired guanine, and a G:C or G:5mC pair selecting hydrogen bond in the central minor groove (65) from the guanine to Asn42. As for the external 5mC, both proximal and distal subunits take part in sequence recognition. The structure explains the surprising tolerance of the BisI family of enzymes to replacements of 5mC with T, since there are no direct hydrogen bonds to the external or internal 5mC that could enforce a distinction between keto- and amino-pyrimidines. In the case of T:A mispairs, the A of the external pair is readily accommodated because the lack of direct interactions. The A of the internal pair can still engage in the hydrogen bond on the major groove site, but the hydrogen bond on the minor groove is lost, which explains the tolerance of T:A pairs and the loss of affinity. In case of G:T mispairs, pseudo Watson–Crick or wobble pairing may explain the T:G tolerance (66).

The central, symmetry breaking base pair is not expected to take part in any interactions at all. Its major groove side is in the vicinity of arginine residues from both subunits (Arg101 in NhoI), but the arginine residues are over 5 Å away, and there are no water molecules to mediate indirect hydrogen bonds. Hence, the structure is consistent with a lack of sequence specificity in the central position, as indicated in the recognition sequence.

### Recognition of the cytosine methyl groups

The cytosine methyl groups are very well defined in the electron density of the NhoI–DNA complex (Figure 7C and D). Hence, we focus on this complex in our discussion of the interaction of the methyl groups with the protein. Due to the ∼34° rotation per residue, the methyl groups of the 5mC bases in the NhoI or Eco15I target sequence stack against the preceding guanine bases in the same DNA strand. Therefore, as for other proteins that do not flip the methylated DNA bases, interaction with the methyl groups is limited to its one side.

In NhoI, the pocket for an external methyl group (GCNG**5mC**) is formed by the proximal enzyme subunit, namely by the Cβ of Ser98 and the side chain of Arg84 (Figure 7C and Supplementary Figure S7), with the side chain of Gln96 only slightly further apart. The interactions with equivalent aminoacids are conserved in the Eco15I_Ntd–DNA complex. The mentioned arginine is also conserved in about half of the other BisI family members and replaced by threonine or valine in the remainder (Supplementary Figure S6, marked in green). The replacement of arginine by histidine drastically reduced the activity of the enzyme. The variant was still able to cleave Xp12 DNA (with 5mC instead of C), albeit roughly 100-fold less effectively than the wild-type protein. Activity on T4gt DNA (with 5hmC instead of C), which is already an inferior substrate for the wild-type protein, was not detectable for the variant (Figure 1E, Supplementary Figure S8).

The pocket for each internal methyl group (G**5mC**NGC) is formed by the side chains. Direct favorable van der Waals contacts are made with two consecutive residues. For both NhoI and Eco15I, these are a histidine and a valine of the distal subunit (His139 and Val140 in NhoI). Inspection of the multiple sequence alignment for the BisI family shows that there is a variability in both positions. The motif for methyl binding can be described as (H/R)(V/I/T/M). Apparently, not only hydrophobic contacts, but the exact amino acids are conserved (Supplementary Figure S6, marked in pink). As expected, the (H/R)(V/I/T/M) motif is not present in PvuII, which is the most similar to the BisI family among non-modification dependent restriction endonucleases. In NhoI, a guanidino group of an arginine residue nearby (Arg101 of the proximal NhoI subunit) also contributes to the internal methyl binding pocket (Figure 7D). Since the (H/R)(V/I/T/M) motif is from the distal protomer, and the arginine from the proximal protomer, the results show that both subunits contribute to each pocket for an internal methyl group. All described interactions of NhoI with the internal methyl groups in DNA are conserved in the Eco15I_Ntd–DNA complex. Here, the relevant residues are His139 and Val140 from the distal subunit and Arg98 from the proximal subunit.

Mutation of His139 to alanine (H139A) in NhoI resulted in a partial impairment of the enzyme activity (Figure 7F). Replacement of Val140 with alanine altered the protein folding and made the protein insoluble (Supplementary Figure S9). In the protein-DNA complex, external and internal methyl groups are fairly well embedded (Figure 7C and D). Nevertheless, there is space to accommodate a hydroxyl group that would be present if the recognition sequence contained 5hmC instead of 5mC.

## Discussion

### Physiological target of the BisI family of restriction endonucleases

Modification-dependent restriction endonucleases have the potential for genetic conflict with conventional restriction modification systems (67). Their widespread distribution suggests that this is a risk worth taking, presumably because the benefits of protection outweigh the risk. In the case of the BisI family of enzymes, however, it is not entirely clear what exactly the enzymes protect against. Based on the greater activity of the enzymes towards methylated than hydroxymethylated DNA, methylated DNA is the more likely physiological target. However, it remains an open question whether the BisI family is directed against densely methylated DNA, created by polymerization of already methylated building blocks (5mdCTP), or against more sparsely methylated DNA that is methylated after DNA polymerization by sequence-specific DNA methyltransferases.

Assuming that the natural target of the BisI family of restriction endonucleases is densely methylated DNA, the enzymes could be directed against phages such as Xp12, which has all cytosines in the genome substituted by 5mC. Xp12 is a *Xanthomonas* phage and among the BisI family enzymes, only one, SmaAUI, from *Stenotrophomonas maltophilia*, comes from *Xanthomonadales*.

Alternatively, the target of the BisI family could also be sparsely methylated phages that have been methylated by host or prophage methyltransferases. Inspection of REBASE (68) shows that methyltransferases with a predicted GCNGC, GCSGC (S stands for C or G), or GCWGC (W stands for A or T) recognition sequence are widespread in bacteria, and particularly abundant in the *Terrabacteria* group, whereas the BisI family enzymes are most enriched in *Pseudomonadota* (Supplementary Figure S10). A few of the listed enzymes (10 out of 381 cases) methylate the internal cytosine bases. For the remainder, it is unknown which cytosine bases of the recognition sequence are methylated. According to REBASE, some of the methyltransferases come with a restriction endonuclease of matching specificity. The orphan methyltransferases include the recently described multi-specific C5 methyltransferase M.BceSV from *Bacillus cereus*, which modifies GCNGC and other similar sites (69), and a *Bacillus anthracis* prophage methyltransferase M.BatI that methylates GCDGC (where D is A, T or G) sites (70). In all cases, the above methyltransferases are expected to introduce no more than two methyl groups in the recognition sequences of BisI family enzymes (except for GCNGCNGC sites). A full methylation of GCNGC, GCSGC or GCWGC recognition sequences could be introduced post-replication by GC specific DNA methyltransferases. Such methyltransferases have indeed been reported (71). However, the only known instances so far are from the *Chlorella* virus (e.g. M.CviPI) and from the viral metagenomics (e.g. M.Vme43I). Therefore, it is unclear whether the enzymes from BisI family could physiologically encounter DNA that has been methylated by the GC specific DNA methyltransferases or frequent cytosine methylases that modify most cytosines except polyC tracks.

### Tolerance for 5mC to T changes

The biochemical experiments in this work and the crystallographic data suggest that the BisI family of enzymes is quite tolerant of changes from 5mC to T in the recognition sequence of the enzymes, regardless of whether the G in the complementary strand is also changed to maintain Watson-Crick pairing (so that the change is 5mC:G to T:A), or retained (so that the 5mC:G pair is changed to a T:G mispair). From a structural perspective, the tolerance for 5mC:G to T:A is explained by the preference of the enzymes for a 5-methyl group, and little or no direct hydrogen bonding with the pyrimidine base (and its Watson–Crick paired base) to distinguish keto- from amino-pyrimidines. Recognition of either 5mC:G or T:A pairs is common in eukaryotic CpG methylation specific proteins (72): CpA methylation in embryonic stem cells can be interpreted as evidence of methyltransferase tolerance for T:A instead of 5mC:G (or C:G) pairs (73,74). Other examples of proteins that accept both 5mC:G and T:A pairs are Kaiso (75), Klf4 (76,77), and the activator protein 1 (AP-1) (78). MeCP2 binds both 5mC:G and T:A, but has a clear affinity preference for the 5mC:G pair, just like Eco15I_Ntd (79). As one would expect on structural grounds, the tolerance for T:A pairs instead of 5mC:G pairs does not extend to proteins that are specific for non-methylated CpG (72). These must discriminate against a methyl group in the 5-position of the pyrimidine ring, and therefore do not tolerate T. While a 5mC:G change to T:A perturbs mostly the hydrogen bonding pattern (and removes an amino group from the central minor groove), a change from a 5mC:G pair to a T:G mispair could be more disruptive. Surprisingly, however, this change is also tolerated by the BisI family. T:G mispairs may be accommodated as wobble pairs (T N3 and O2 hydrogen bonded with G O6 and N1, respectively). Alternatively, the T:G mispairs could from pseudo Watson-Crick pairs (T N3 and O2 hydrogen bonded with G N1 and N2, respectively), after N1 deprotonation of the guanine to the anion (66). In the absence of crystal structures containing T:G mispairs in the recognition sequence, we cannot distinguish between the two possibilities.

In contrast to some of the eukaryotic proteins, which bind 5mC:G and T:A pairs comparably well, Eco15I_Ntd, and likely also other Bis family members, bind 5mC:G much tighter than T:A. Hence, the likely physiological substrates of the BisI family are DNAs with densely methylated recognition sequences, as originally reported (34,35).

### Contribution of external and internal methyl binding pockets

The SPR data show that the affinity of Eco15I_Ntd (and by implication, of other BisI family members) for its target increases with the number of methyl groups in the recognition sequence and decreases with salt concentration. We attribute the two effects to the hydrophobic effect, and screening of favorable electrostatic interactions between enzyme and DNA backbone, respectively. Internal and external methyl binding pockets contribute roughly equally. Removal of one methyl group from the recognition sequence decreases binding by less than one order of magnitude in low salt (50 mM), but by two to three orders of magnitude in higher salt (150 mM). This finding indicates that a methyl group in the target contributes more to enzyme binding in high than low salt. We interpret this finding as a manifestation of the ‘salting out’ effect, which results from the higher penalty of exposing hydrophobic surfaces in high salt.

### Tunability of the required number of methyl groups for DNA cleavage by the salt concentration

Based on experiments at a fixed salt concentration, it has been reported that members of the BisI family require an (enzyme dependent) minimum number of methyl groups in their target sequences (24). Our data show that this minimum number of methyl groups is dependent on conditions and can be tuned by altering the salt concentration. The higher the salt, the more shielded and hence weaker the sequence- and modification independent interactions with the phosphodiester backbone of the DNA are. Conversely, the free energy advantage from the burial of methyl groups upon complex formation grows with the salt concentration (compare *K*_D_ changes from omission of one methyl group in low and high salt in Figure 3). The two effects come together to make methyl groups in the target both more needed and more effective to promote binding in high salt conditions. The requirement of 5mC in GCNGC can be increased to four for Eco15I_Ntd efficient cleavage in high salt condition (400 mM KCl; Figure 2A). Interestingly, it was reported that Eco15I (WT) required fewer modifications in the substrate than NhoI (34), in agreement with our findings for lower salt concentrations (Figure 2). Hence, Eco15I may have evolved to also restrict less densely modified DNA, in addition to the densely modified DNA that is cleaved as well by NhoI. The ability to adjust the number of methyl groups required for DNA cleavage by salt concentration may be exploited in biotechnological applications of the BisI family of enzymes.

### Recognition of 5mC (and 5-hydroxymethylcytosine) in the context of dsDNA

5-methyl or 5-hydroxymethyl cytosine substituents are located on the external major groove, in the vicinity of the phosphodiester backbone of DNA, which partially shields access to them. By contrast, the same groups attached to adenine would be placed in the central major groove, in the region of the DNA bases most accessible to enzymatic probes (80). Moreover, unlike adenine methyl groups in ApT context, C5-methyl or hydroxymethyl groups cannot interact with each other in any sequence context, hence DNA deformations from steric conflicts of methyl or hydroxymethyl groups cannot be exploited (23). It is therefore not surprising that recognition of C5-methyl or hydroxymethyl groups in the context of dsDNA, without nucleotide flipping, is rare, at least in prokaryotes. A good example of selection for methyl and hydroxymethyl groups in restriction biology is NEco (22), the methylation sensing domain of EcoKMcrA (21). Here too, hydrophobic residues are involved in recognition, however their identity is different. In NEco, the main contributor to the pocket is the indole ring of a tryptophan residue, whereas neither of the two residues of the (H/R)(V/I/T/M) methyl/hydroxymethyl pocket motif in the BisI family is a tryptophan. The important contribution of an amino acid main chain to one of the methyl/hydroxymethyl binding pockets is a common theme in the BisI family and in NEco.

In eukaryotes, detection of 5-methyl groups (and in some cases also 5-hydroxymethyl groups) without flipping, in the context of dsDNA, is more common (81). Examples are MBDs (82), MeCP2 (83), and some zinc finger proteins including Zfp57 (84) and Kaiso (75). All of these enzymes have similar folds and are likely phylogenetically related. A recurring motif in the structures of these complexes with methylated DNA is the stacking of a methyl group against the guanidino group of an arginine residue (see Supplementary Figure S13 of Slyvka *et al.* (22)). A similar interaction of the external methyl groups of the recognition sequence with arginine residues (Arg84) is seen in the NhoI–DNA complex. NhoI Arg84 is only moderately conserved in the BisI family. However, even the conservative substitution to histidine drastically reduced the activity of the variant against methylated DNA. For NhoI with 5hmC bases in the target, the variant did not retain detectable activity in our assays at all.

In previous work, we have shown how PD-(D/E)XK domains can acquire specificity for methylated adenines in the case of DpnI and related enzymes (23). Here, we complement the earlier work by demonstrating how the same endonuclease family has acquired specificity for methylated cytosine bases. The methyl group detection strategies have in common that the methyl groups are detected in the context of double-stranded DNA, without flipping. However, they are otherwise quite different, as would be expected, given the very different placement of adenine and cytosine methyl groups in the major groove of DNA (85). Adenine methyl groups in ApT context are spatially close, and are therefore recognized together. In contrast, methyl groups in the recognition sequence of BisI family targets are all recognized separately, in a manner reminiscent of methyl detection by eukaryotic 5mC specific proteins. Given the large number of restriction enzymes in the BisI family, this appears to be an evolutionarily successful strategy (Supplementary Figure S10).

## Supplementary Material

gkae634_Supplemental_File

## Data Availability

The data underlying this article are available in PDB and can be accessed with accession numbers: 8Q5M (Eco15I_Ntd), 8Q5N (NhoI), 8Q5O (Eco15I_Ntd-DNA) and 8RPX (NhoI-DNA). The corresponding diffraction images are available under DOIs: https://doi.org/10.18150/FKXIGF (Eco15I_Ntd), https://doi.org/10.18150/TMDAW5 (NhoI), https://doi.org/10.18150/E94VAO (Eco15I_Ntd-DNA) and https://doi.org/10.18150/MKQD3P (NhoI-DNA).

## Notes

Present address: Marek Wojciechowski, Plant Breeding and Acclimatization Institute – National Research Institute, Błonie, Poland.

Present address: Asgar Abbas Kazrani, Institute of Genetics and Molecular and Cellular Biology, University of Strasbourg, Illkirch, France.
