# Supplementary material for: Structural analysis of the BisI family of modification dependent restriction endonucleases
Source: Nucleic Acids Res. 2024 Jul 23;52(15):9103–18. doi: 10.1093/nar/gkae634 (PMC11347163; doi:10.1093/nar/gkae634)

**Supplementary Material**

**Suppl. Tables S1-S4**

**Suppl. Figures S1-S10**

# Structural analysis of the BisI family of modification dependent restriction endonucleases


Katarzyna Szafran[1,#], Dominik Rafalski[1, 2, #], Krzysztof Skowronek[1], Marek Wojciechowski[1, a], Asgar Abbas Kazrani[1, b], Mirosław Gilski[3,4], Shuang-yong Xu[5], Matthias Bochtler[1,2]

[1]*International Institute of Molecular and Cell Biology, Warsaw, Poland*

[2]*Institute of Biochemistry and Biophysics, Polish Academy of Sciences, Warsaw, Poland*

[3] *Faculty of Chemistry, Adam Mickiewicz University, Poznan*

[4] *Institute of Bioorganic Chemistry, Polish Academy of Sciences, Poznan, Poland*

[5] *New England Biolabs, Ipswich, USA*

[a]*Current address: Plant Breeding and Acclimatization Institute – National Research Institute, Błonie, Poland*

[b] *Current address: Institute of Genetics and Molecular and Cellular Biology, University of Strasbourg, Illkirch, France*

[#]*Equal contribution*

*Correspondence to mbochtler@iimcb.gov.pl*

## SUPPLEMENTARY TABLES

**Table S1.** Amino acid sequences of wild-type proteins used in the study.

| Name | Sequence |
|---|---|
| Eco15I | MKNNHLTSYDWLNALNNLELLSLHSEILTQLRSRGVIRTKNNPVGDYAEWLVSNALGMTLL<br>SNSSAGADAIDADGLKVQIKARRVTPDNPSRQLSALRNYEAADFDYLIAVIFDETYNILDA<br>YKIPHEVIRDYARHSDHVNAHIVNLKGAILTDPRVSSIKEDLIVRSSASVNEAAMQTLPPE<br>PVEEVLNQPEKITSSVTLVSLLKAIGMECFVNYYHHFADSNLPSAYIIEQMHSREGYTEKS<br>CRSRLSKARKVIREGLSIEALALIADSERMQDSVRNDALKLISVLE |
| Eco15I_Ntd | SYDWLNALNNLELLSLHSEILTQLRSRGVIRTKNNPVGDYAEWLVSNALGMTLLSNSSAGA<br>DAIDADGLKVQIKARRVTPDNPSRQLSALRNYEAADFDYLIAVIFDETYNILDAYKIPHEV<br>IRDYARHSDHVNAHIVNLKGAILTDPRVSSIKEDLIVRSSASVNEAAMQTL |
| Eco15I_12A | ALNNLELLSLHSEILTQLRSRGVIRTKNNPVGDYAEWLVSNALGMTLLSNSSAGADAIDAD<br>GLKVQIKARRVTPDNPSRQLSALRNYEAADFDYLIAVIFDETYNILDAYKIPHEVIRDYAR<br>HSDHVNAHIVNLKGAILTDPRVSSIKEDLIVRSSASVNEAAMQTL |
| Eco15I_Ctd | PPEPVEEVLNQPEKITSSVTLVSLLKAIGMECFVNYYHHFADSNLPSAYIIEQMHSREGYT<br>EKSCRSRLSKARKVIREGLSIEALALIADSERMQDSVRNDALKLISVLE |
| NhoI | MSRPPSYAGDMNLENLTTRELLAVSRASLRELKRRGVIRSGNAPAGDYAELLVQRATDGEL<br>ANASQKSWDIRTTEGDRLQVKARVITDEHANGERQLSTIRSWDFDAAVIVLFDDNFRVWRA<br>ARVPAAIMKEAAYYSQHVRGYTVYAKDALLNHSEVEDWTEQLRSVEQ |

**Table S2.** Primers used for site-directed mutagenesis of Eco15I_Ntd and NhoI. Codons for the amino acid substitutions are marked in bold.

| Name | Sequence | Modified construct |
|---|---|---|
| E49A_For | GTTGGTGACTACGCG**GCA**TGGCTGGTTTCTAAC | Eco15I_Ntd |
| E49A_Rev | GTTAGAAACCAGCCA**TGC**CGCGTAGTCACCAAC | |
| D69A_For | GGTGCG**GCC**GCGATC | |
| D69A_Rev | GATCGC**GGC**CGCACC | |
| Q79A_For | GTCTGAAAGTT**GCG**ATCAAAGCG | |
| Q79A_Rev | CGCTTTGAT**CGC**AACTTTCAGAC | |
| K81A_For | GTTCAGATC**GCA**GCGCGTC | |
| K81A_Rev | GACGCGC**TGC**GATCTGAAC | |
| H139A_For | GTACTACTCTCAG**GCC**GTTCGTGGTTAC | NhoI |
| H139A_Rev | GTAACCACGAAC**GGC**CTGAGAGTAGTAC | |
| V140A_For | CTACTCTCAGCAC**GCT**CGTGGTTACACC | |
| V140A_Rev | GGTGTAACCACG**AGC**GTGCTGAGAGTAG | |

**Table S3.** Oligonucleotides used in SPR experiments, activity assays and EMSA; **X** = 5mC; [Btn] = biotin

| Name | Sequence | Application | Symbol |
|---|---|---|---|
| 10/01 | [Btn]CGGTTGAG**X**AGCACCTCAGA<br>GCCAACT**X**GTCGTGGAGTCT | Activity assay | 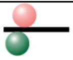 |
| 10/11 | [Btn]CGGTTGAG**X**AGCACCTCAGA<br>GCCAACT**X**GT**X**GTGGAGTCT | Activity assay, SPR | 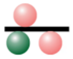 |
| 11/01 | [Btn]CGGTTGAG**X**AG**X**ACCTCAGA<br>GCCAACT**X**GTCGTGGAGTCT | SPR | 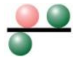 |
| 11/11 | [Btn]CGGTTGAG**X**AG**X**ACCTCAGA<br>GCCAACT**X**GT**X**GTGGAGTCT | Activity assays, SPR, EMSA | 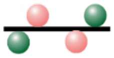 |
| EA | CGGTTGAA**X**AG**X**ACCTCAGA<br>GCCAACT**T**GT**X**GTGGAGTCT | Activity assay | 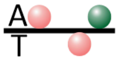 |
| IA | CGGTTGAG**X**AA**X**ACCTCAGA<br>GCCAACT**X**GT**T**GTGGAGTCT | Activity assay | 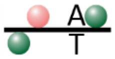 |
| EG | CGGTTGAG**X**AG**X**ACCTCAGA<br>GCCAACT**T**GT**X**GTGGAGTCT | Activity assay | 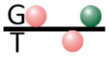 |
| IG | CGGTTGAG**X**AG**X**ACCTCAGA<br>GCCAACT**X**GT**T**GTGGAGTCT | Activity assay | 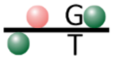 |
| E2AM | CGGTTGAA**X**AG**T**ACCTCAGA<br>GCCAACT**T**GT**X**ATGGAGTCT | Activity assay | 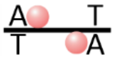 |
| E2GM | CGGTTGAG**X**AG**T**ACCTCAGA<br>GCCAACT**T**GT**X**GTGGAGTCT | Activity assay | 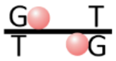 |
| EAT | CGGTTGAG**T**AA**X**ACCTCAGA<br>GCCAACT**X**AT**T**GTGGAGTCT | Activity assay, EMSA | 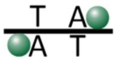 |
| EGT | CGGTTGAG**T**AG**X**ACCTCAGA<br>GCCAACT**X**GT**T**GTGGAGTCT | Activity assay, EMSA | 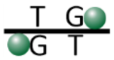 |
| ATT5 | CGGTTGAA**X**AA**T**ACCTCAGA<br>GCCAACT**T**GT**T**ATGGAGTCT | Activity assay, EMSA | 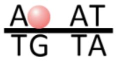 |
| GTT5 | CGGTTGAG**X**AG**T**ACCTCAGA<br>GCCAACT**T**GT**T**GTGGAGTCT | Activity assay, EMSA | 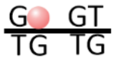 |

**Table S4. Data collection and refinement statistics.** Statistics for the highest-resolution shell are shown in parentheses.

| | Eco15I_Ntd-DNA | Eco15I_Ntd-Apo | NhoI-DNA | NhoI-Apo |
|---|---|---|---|---|
| **Resolution range (Å)** | 48.00-2.33 (2.41-2.33) | 43.04-2.66 (2.76-2.66) | 46.81- 1.81 (1.88-1.81) | 46.25- 2.65 (2.75-2.65) |
| **Space group** | $P\ 3_2\ 2\ 1$ | $P\ 2_1\ 2_1\ 2_1$ | $P\ 2_1\ 2_1\ 2$ | $P\ 1\ 2_1\ 1$ |
| **Cell constans** | 96.00 96.00 96.31 90.00 90.00 120.00 | 65.69 109.71 113.95 90.00 90.00 90.00 | 114.64 167.06 56.51 90.00 90.00 90.00 | 38.48 58.76 75.59 90.00 97.23 90.00 |
| **Total reflections** | 438545 (45434) | 194286 (18640) | 1310702 (131576) | 65269 (6535) |
| **Unique reflections** | 22349 (2202) | 24304 (2390) | 99666 (9866) | 9793 (990) |
| **Completeness (%)** | 99.77 (99.68) | 99.77 (99.92) | 99.83 (99.73) | 99.17 (99.60) |
| **$I/\sigma_I$** | 20.04 (4.33) | 29.39 (2.08) | 18.05 (2.03) | 14.57 (2.40) |
| **Wilson B-factor ($Å^2$)** | 51.05 | 83.09 | 36.60 | 54.22 |
| **$CC_{1/2}$** | 1.00 (0.961) | 1 (0.790) | 0.998 (0.911) | 0.997 (0.852) |
| **$R_{merge}$ (%)** | 10.96 (129.40) | 3.84 (89.83) | 7.15 (112.50) | 11.27 (83.02) |
| **$R_{meas}$ (%)** | 11.27 (132.70) | 4.11 (96.26) | 7.46 (117.00) | 12.24 (90.07) |
| **$R_{pim}$ (%)** | 2.59 (28.99) | 1.45 (34.19) | 2.09 (31.72) | 4.71 (34.48) |
| **$R_{work}$ (%)** | 26.41 (42.21) | 25.90 (30.50) | 20.01 (33.37) | 22.16 (28.42) |
| **$R_{free}$ (%)** | 27.60 (43.45) | 28.43 (33.05) | 24.24 (37.59) | 27.05 (27.62) |
| **Number of non-hydrogen atoms** | 2686 | 4266 | 8614 | 2507 |
| **macromolecules** | 2660 | 4218 | 8063 | 2448 |
| **ligands** | 2 | 0 | 37 | 18 |
| **solvent** | 24 | 48 | 514 | 41 |
| **Protein residues** | 300 | 535 | 796 | 306 |
| **RMS (bonds) (Å)** | 0.010 | 0.007 | 0.008 | 0.004 |
| **RMS (angles) ($^o$)** | 1.40 | 0.93 | 1.02 | 0.80 |
| **Ramachandran favoured (%)** | 91.26 | 97.23 | 98.73 | 97.67 |
| **Ramachandran allowed (%)** | 8.74 | 2.38 | 1.02 | 2.33 |
| **Ramachandran outliers (%)** | 0.00 | 0.40 | 0.25 | 0.00 |
| **Rotamer outliers (%)** | 5.88 | 0.87 | 2.96 | 2.78 |
| **Clashscore** | 25.62 | 4.38 | 4.48 | 7.14 |
| **Average B-factor ($Å^2$)** | 135.0 | 100.8 | 53.0 | 74.5 |
| **PDB accession code** | 8Q5O | 8Q5M | 8RPX | 8Q5N |

## SUPPLEMENTARY FIGURES

**Figure S1**. **Schematic representation of the proteins used in this study and design of the shortest active form of Eco15I (A, B)** 6 or 12 N-terminal amino acids of Eco15I N-terminal domain were eliminated. The cleavage assay with recombinant proteins confirmed that only protein Eco15I_Ntd is active. Moreover, full-length NhoI was the subject of this study **(A). (C)** The activity of full-length Eco15Iand Eco15I_Ntd were also compared. In **(B)** 30 ng of 550bp PCR product was incubated with 0.8-8pmol of Eco15I_Ntd or 0.9-9 pmol 12A for 40 minutes at 37 ºC. In **(C)** and 10 pmols of 20 bp long dsDNA were incubated with 5 pmols of protein for 1h at 37°C. Reaction conditions varied with salt concentration. The orientation of methyl groups is depicted schematically – internal 5mC is marked in pink and external 5mC is marked in green. The gels show representative data for at least 3 repeats.

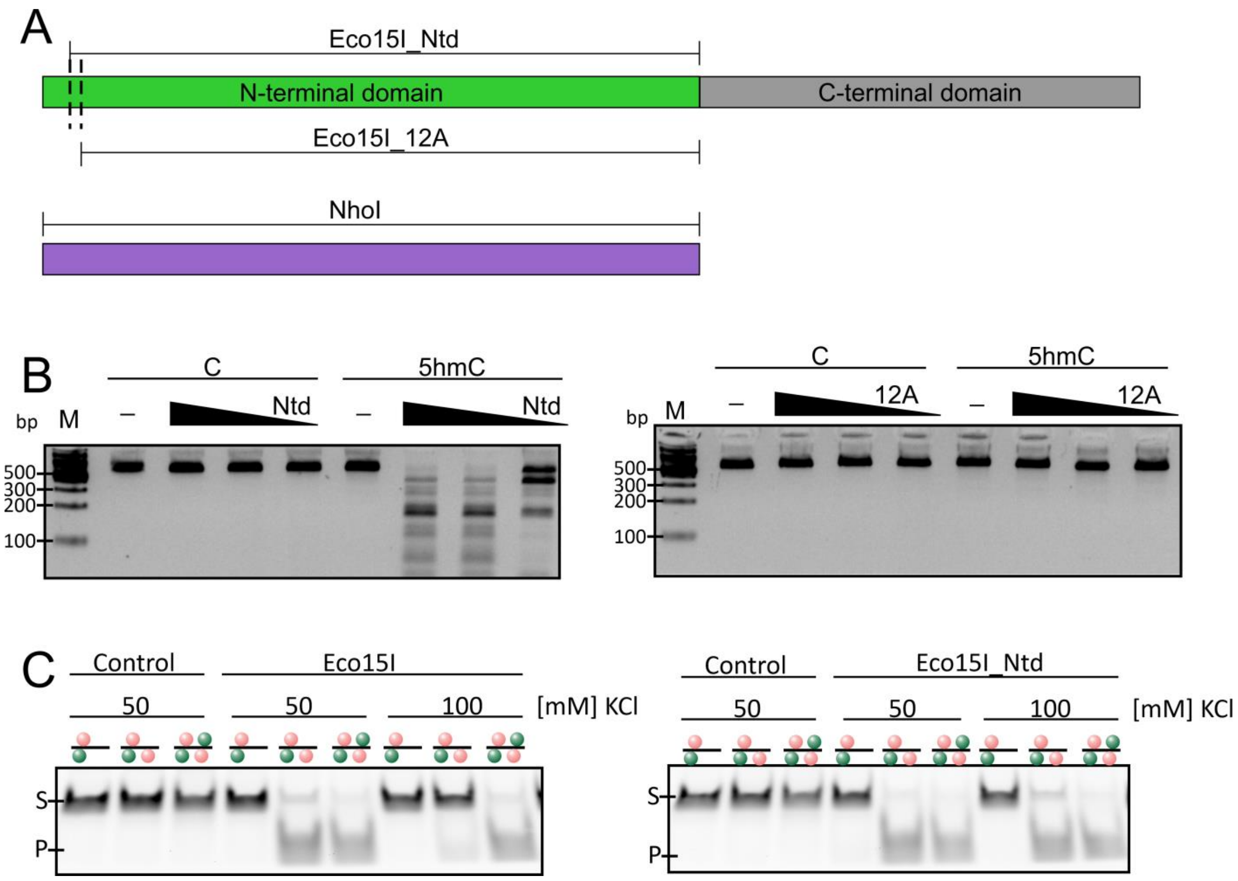

**Figure S2. SPR response curves for the interaction of Eco15I_Ctd (A) and Eco15I_Ntd (B) with dsDNA oligonucleotide containing fully methylated target sequence.** DNA was immobilized in the sensor at concentration of 2nM. Concentration of injected protein is marked in colors as indicated in the figure legend. Experiments were carried out in a buffer containing 50 mM KCl.

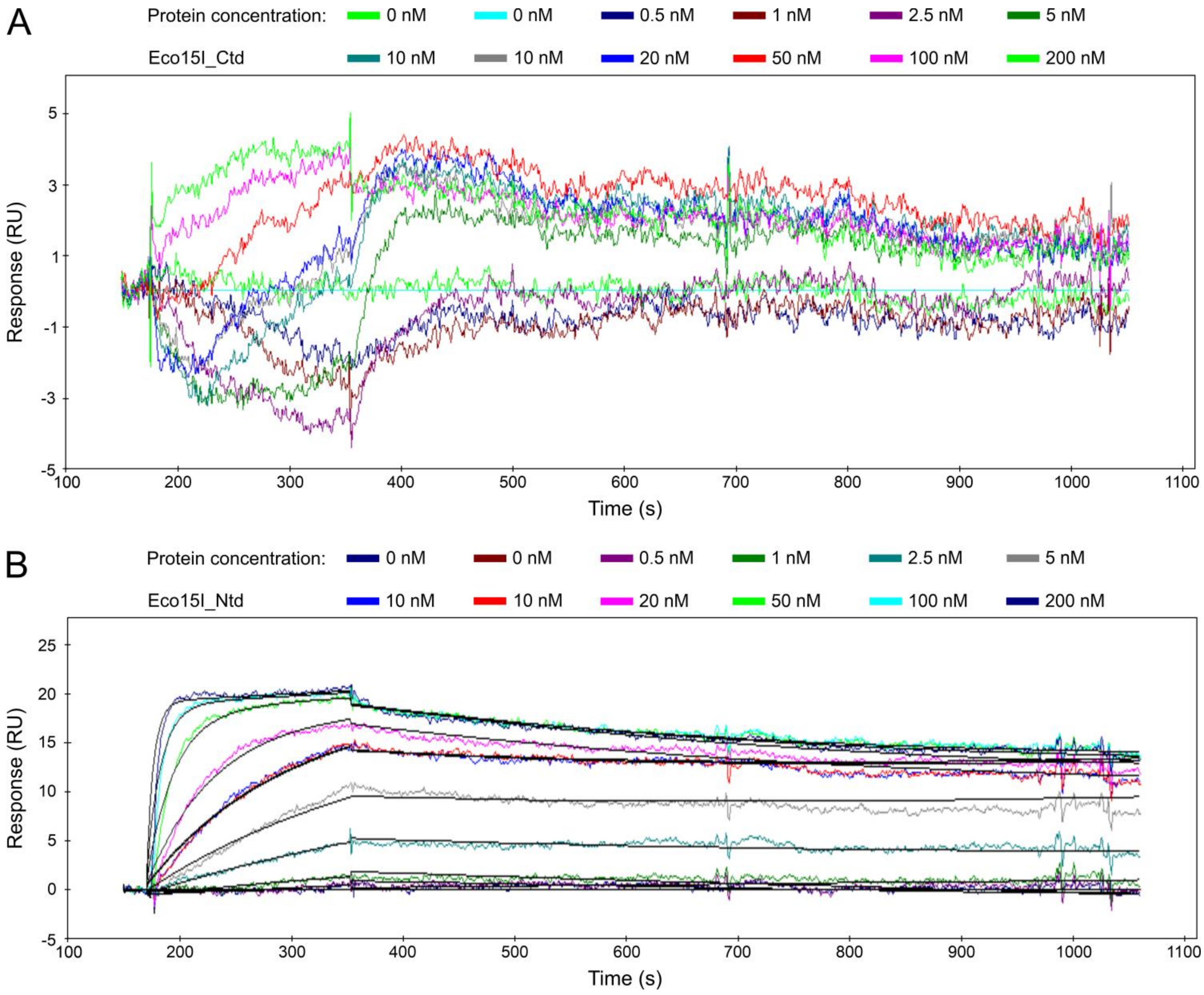

**Figure S3. Eco15I_Ntd activity and binding to various substrates.** Substrates contained a variable number of methyl groups and base substitutions within the enzyme recognition sequence. The orientation of methyl groups is depicted schematically. Internal and external 5mC bases are shown as pink and green balls, respectively. **(A)** Effect of the replacement of 5mC bases in the target sequence by T bases, paired with either A or G. **(B)** Comparison of substrates with two 5mC bases (and a substrate with four 5mC bases as a control). **(C)** Comparison of the activity of Eco15I_Ntd WT and mutants. In all cleavage assays (panels A, B, C) 10 pmols of DNA were incubated with 5 pmols of protein for 40 min at 37 °C, and digestion mixes were then analyzed by native polyacrylamide (PAA) electrophoresis. **(D)** Electrophoretic mobility shift assay (EMSA) to compare affinities of substrates with 5mC:G, T:G and T:A pairs. 1 pmol of DNA was incubated with increasing amounts (indicated below in pmol) of Eco15INtd for 30 min on ice. Theresults were visualized by autoradiography. All panels show representative data for at least 3 repeats.

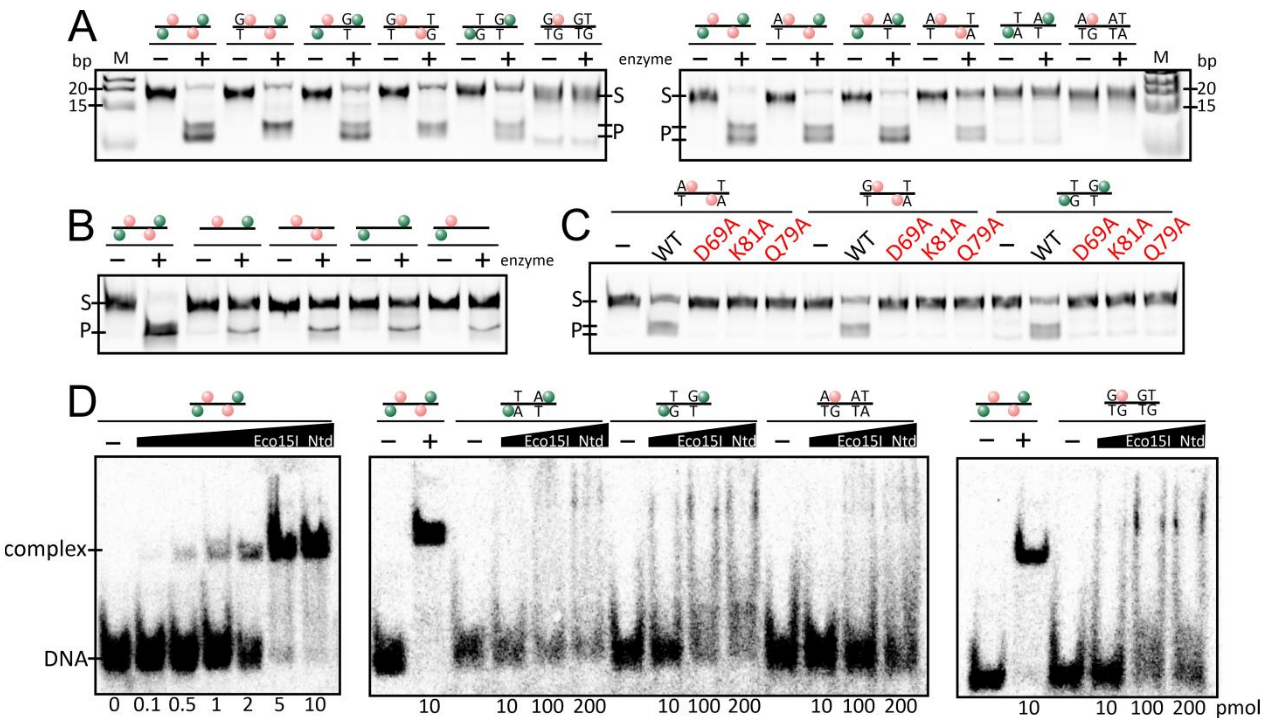

**Figure S4. NhoI activity for various concentrations of the enzyme.** Oligonucleotides with the NhoI recognition sequence and 2-4 methyl groups were cleaved with increasing amounts of enzymes, and the reaction products were analyzed by native polyacrylamide (PAA) gels. The orientation of methyl groups is depicted schematically – internal 5mC is marked in pink and external 5mC is marked in green. 10 pmols of DNA were incubated with 5 pmols of protein for 1h at 37°C. The gel shows representative data for 3 repeats.

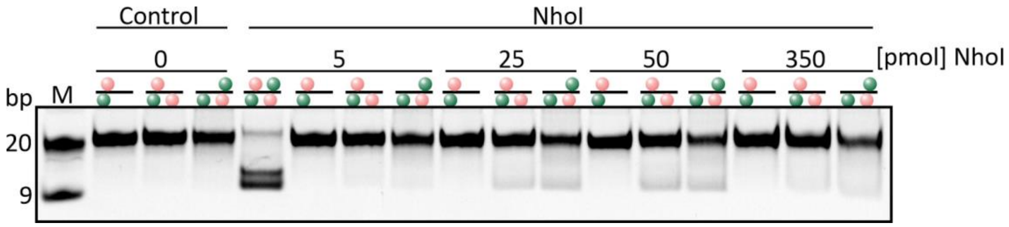

**Figure S5. Fitting of sensor response vs. concentration plot to the basic steady state affinity model, Biacore S200.** The orientation of methyl groups is shown schematically. Experiments were carried out in a buffer containing either 50 mM or 150 mM KCl. Insets indicate the Kd in molar units, and "SE" stands for "standard error".

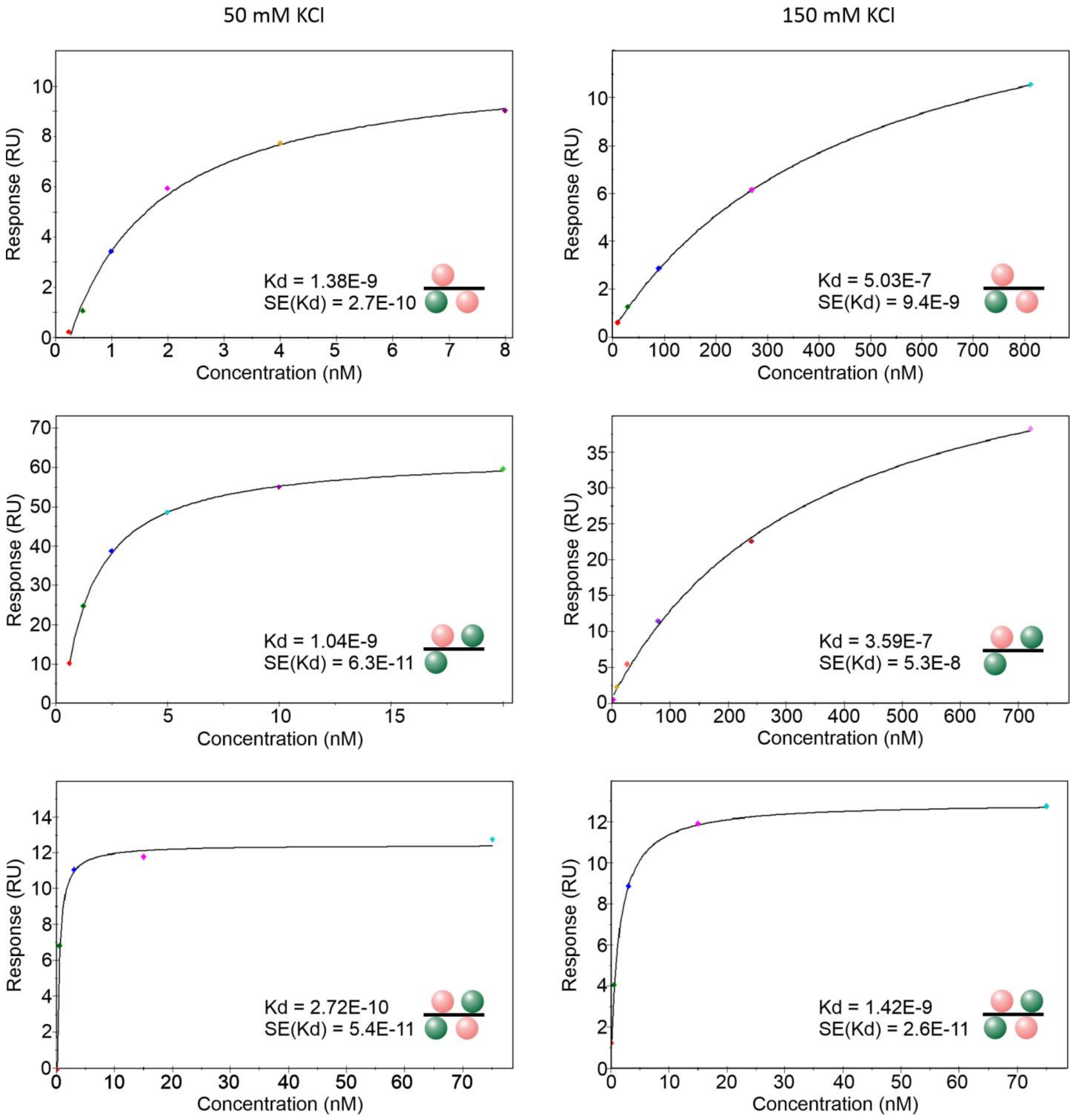

**Figure S6. Multiple sequence alignment of BisI and its homologs.** Residues responsible for divalent cations ($Mg^{2+}$) coordination are marked in blue, external methyl binding – in green and internal methyl binding – in pink.

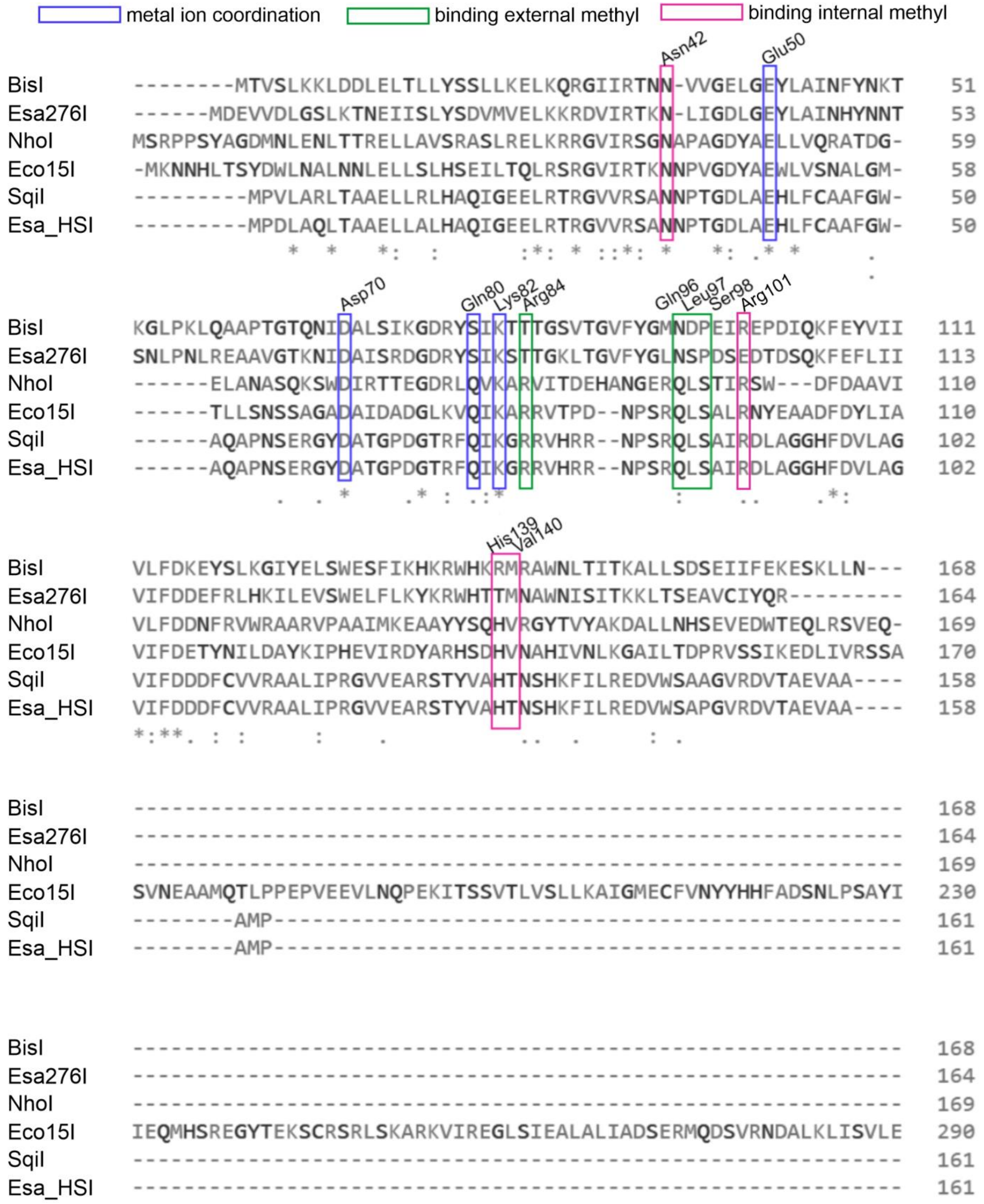

**Figure S7. Space filling representation of the binding pockets for external and internal methyl groups.** Atoms are represented as balls with their van der Waals radii, and colored according to their atom type (carbons of the protein are green, and those of the 5mC are yellow). For the methyl group, a van der Waals radius of 2.0 Å was taken.

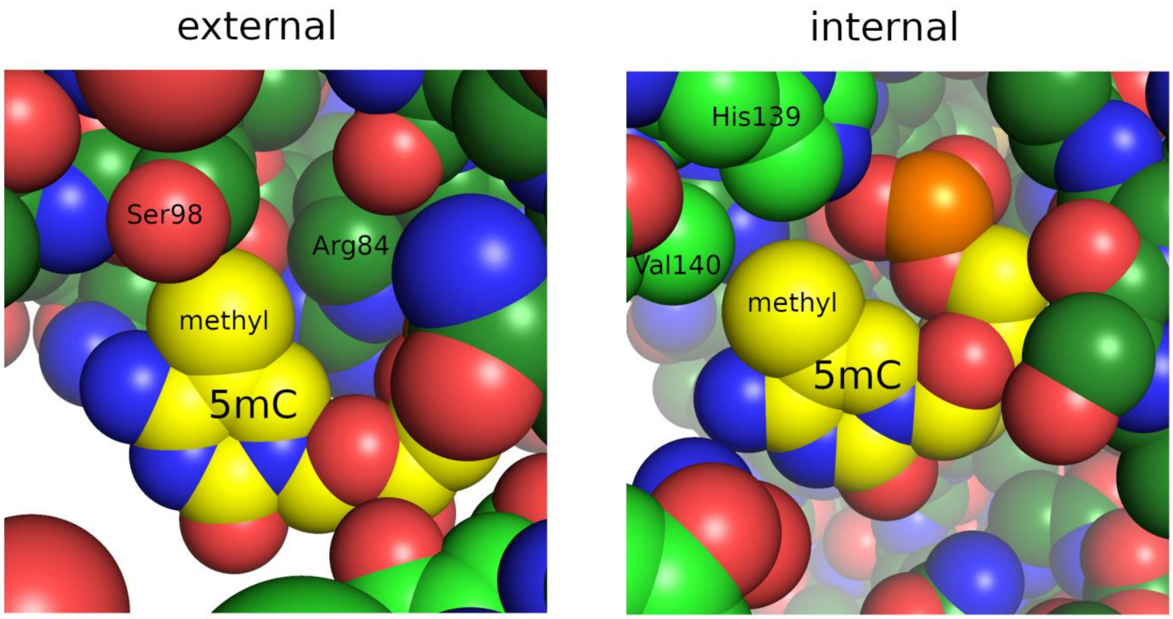


**Figure S8. Substrate preference of NhoI mutant R84H.** Digestions were performed with 200 ng of phage DNA and 150 ng of 3kb 5hmC-PCR product and 0.02–2 µg of enzyme at 37 ºC, for 1 h. The gel shows representative data for 3 repeats.

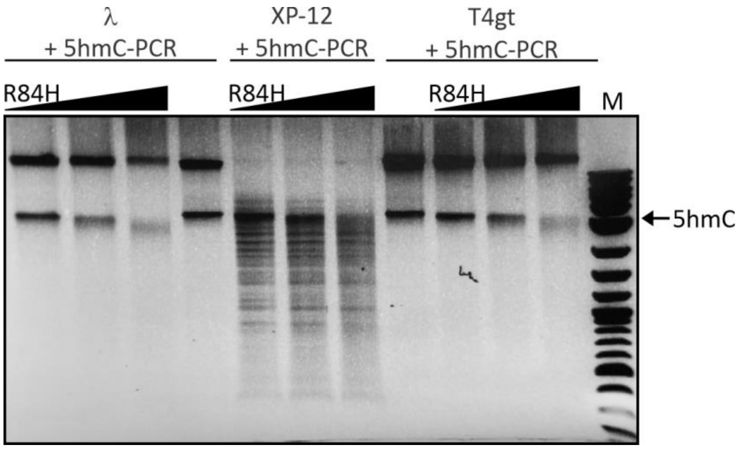

**Figure S9. Solubility test of NhoI_V140A.** The protein fractions obtained after lysis. T - total lysate, S - soluble fraction, P - insoluble fraction. The gel shows representative data for 3 repeats.

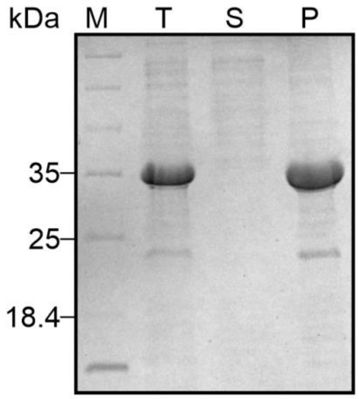


**Figure S10. Comparison of the origin of BisI family enzymes and methyltransferases generating cognate target sequences.** Methyltransferases with GCNGC, GCSGC or GCWGC specificity are marked in blue. BisI family members of activity confirmed *in vitro* are marked in orange and putative BisI family members are marked in red. Phylogeny was done using taxopy (https://github.com/apcamargo/taxopy) and the diagram was drawn with graphlan (https://github.com/biobakery/graphlan).

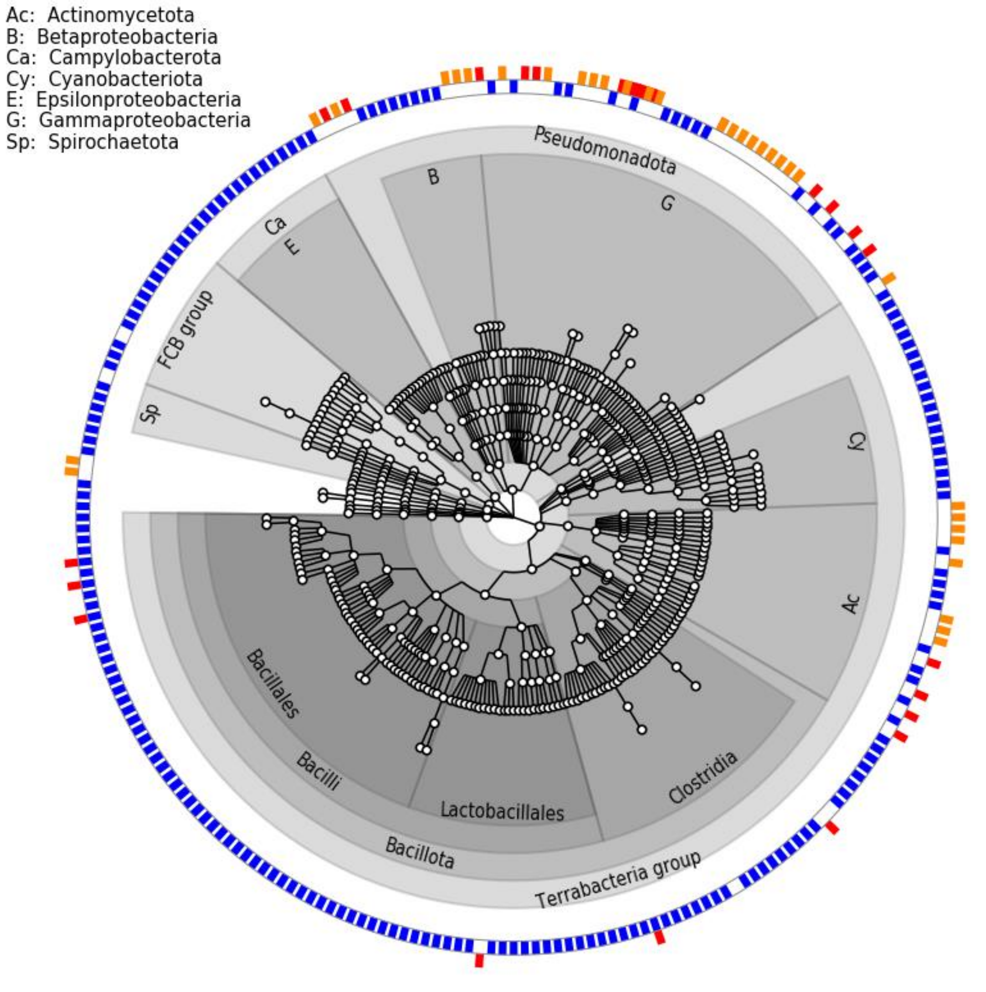

Supplement: gkae634_Supplemental_File [file gkae634_supplemental_file.pdf]
